# From theory to experiment: transformer-based generation enables rapid discovery of novel reactions

**DOI:** 10.1186/s13321-022-00638-z

**Published:** 2022-09-02

**Authors:** Xinqiao Wang, Chuansheng Yao, Yun Zhang, Jiahui Yu, Haoran Qiao, Chengyun Zhang, Yejian Wu, Renren Bai, Hongliang Duan

**Affiliations:** 1 Artificial Intelligence Aided Drug Discovery Institute, College of Pharmaceutical Sciences, Zhejiang University of Technology, Hangzhou, 310014 People’s Republic of China; 2 State Key Laboratory of Drug Research, Shanghai Institute of Materia Medica (SIMM), Chinese Academy of Sciences, Shanghai, 201203 China; 3 College of Pharmacy, School of Medicine, Hangzhou Normal University, Hangzhou, People’s Republic of China; 4 Key Laboratory of Elemene Class Anti-Cancer Chinese Medicines, Engineering Laboratory of Development and Application of Traditional Chinese Medicines, Collaborative Innovation Center of Traditional Chinese Medicines of Zhejiang Province, Hangzhou Normal University, Hangzhou, People’s Republic of China; 5 College of Mathematics and Physics, Shanghai University of Electric Power, Shanghai, 201203 People’s Republic of China

**Keywords:** Deep learning, Heck reactions, Reaction generation

## Abstract

Deep learning methods, such as reaction prediction and retrosynthesis analysis, have demonstrated their significance in the chemical field. However, the de novo generation of novel reactions using artificial intelligence technology requires further exploration. Inspired by molecular generation, we proposed a novel task of reaction generation. Herein, Heck reactions were applied to train the transformer model, a state-of-art natural language process model, to generate 4717 reactions after sampling and processing. Then, 2253 novel Heck reactions were confirmed by organizing chemists to judge the generated reactions. More importantly, further organic synthesis experiments were performed to verify the accuracy and feasibility of representative reactions. The total process, from Heck reaction generation to experimental verification, required only 15 days, demonstrating that our model has well-learned reaction rules in-depth and can contribute to novel reaction discovery and chemical space exploration.

**Supplementary Information:**

The online version contains supplementary material available at 10.1186/s13321-022-00638-z.

## Introduction

Organic synthesis, an important approach for producing novel and complex compounds, is crucial to the pharmaceutical industry. Traditionally, the discovery of new reactions relies on the “chemical intuition” of chemists, requiring extensive experience and plenty of time. Thus far, although researchers have achieved steady progress over the past few decades, only a miniscule fraction of the reaction space has been explored owing to the complexity of reactions.

With the advancement of computer technology, scientists have used machine learning to solve diverse chemical challenges [1–4]. In particular, artificial intelligence (AI) technology significantly contributes to the field of chemical reactions including reaction prediction [5, 6] and retrosynthesis analysis [7–10]. The first computational program category was based on a reaction template, which could perform retrosynthetic analysis or reaction prediction based on hand-coded rules or automatically extracted reaction templates. For instance, CAMEO, a template-based chemical reaction prediction program, was proposed by Salatin et al*.* in 1980 [11]. For retrosynthetic analysis, Coley et al*.* predicted reactants with a templated-based model based on molecular similarity. However, template-based methods have the limitation of only inferring reactions covered by training templates [12]. To overcome this limitation, Yan et al*.* proposed the templates composed with basic template blocks extracted from training templates and achieved a 5.2% improvement [13]. Moreover, Wan et al*.* proposed that the reaction space can be factorized into molecular space and reaction template space, and they attempted to improve the efficiency of reaction space exploration using a smaller reaction template space, achieving a top-1 accuracy of 72.5% in retrosynthesis prediction [14].

On the other hand, template-free methods are focused on directly generating reactants via deep learning without requiring additional feature extraction. Currently, the models for those two tasks can be divided into graph-based and text-based classes [15–18]. The former calculates based on graph structure and the latter uses SMILES. The representative model is the seq2seq model, proposed by Liu et al*.*, which formulates the retrosynthesis prediction as a sequence translation task [19]. Another powerful model is the Transformer-based Molecular Transformer proposed by Schwaller et al*.* in 2019 for reaction prediction [20]. Notably, reaction prediction and retrosynthesis planning are both based on the process that accepts a part of chemical reactions as the input and the remaining reactions is produced as the output.

Inspired by the performance of AI approaches in reaction prediction and retrosynthesis analysis, we put forward a question: is it feasible for AI to generate entirely new reactions similar to the given reactions? It may be similar to the case of de novo molecular generation using deep learning [21, 22]. Sequence-based models are pivotal in molecular generation with SMILES strings represented because of their excellent performance of text, such as poems [23]. Therefore, we attempt to apply the sequence-based model to generate new reactions and naming it “reaction generation”. Additionally, we hope that our work can provide new ideas in exploring the chemical reactions.

Although Bort et al*.* have used recurrent neural networks (RNNs) and condensed graph of reactions to investigate the generation of Suzuki reaction [24], the assessment of the executive experiment remains lacked. Unlike the RNNs they used, we introduced a more powerful SMILE-based model known as the Transformer model. In recent years, the transformer model, proposed by Google for solving machine translation tasks, has been among the most frequently used neural networks which adopts encoder-decoder framework [25]. Compared with the models used in previous works, such as RNNs or long short-term memory (LSTM), this novel model is based solely on attention mechanisms. In recent years, this model has attracted significant attention in the field of chemistry and has been used to multiple tasks in processing reactions [26]. For instance, Vaucher et al*.* achieved the prediction of the experimental process [27]. Moreover, several forms of the transformer models have been derived, such as the Transformer-XL model, to overcome its shortcomings [28]. It enables learning dependency beyond a fixed-length without disrupting temporal coherence, which is a limitation of the Transformer model.

Selecting an appropriate reaction is conducive for reaction generation. In this study, we selected the Heck reaction, a typical carbon–carbon coupling reaction, as a representative experiment to enhance the convenience of drug discovery for its widespread application in alkene synthesis. Its discoverer, R. F. Heck, was awarded the Nobel Prize for this significant contribution [29]. The mechanism is shown in Additional file 1: Fig. S1.

**Fig. 1:**
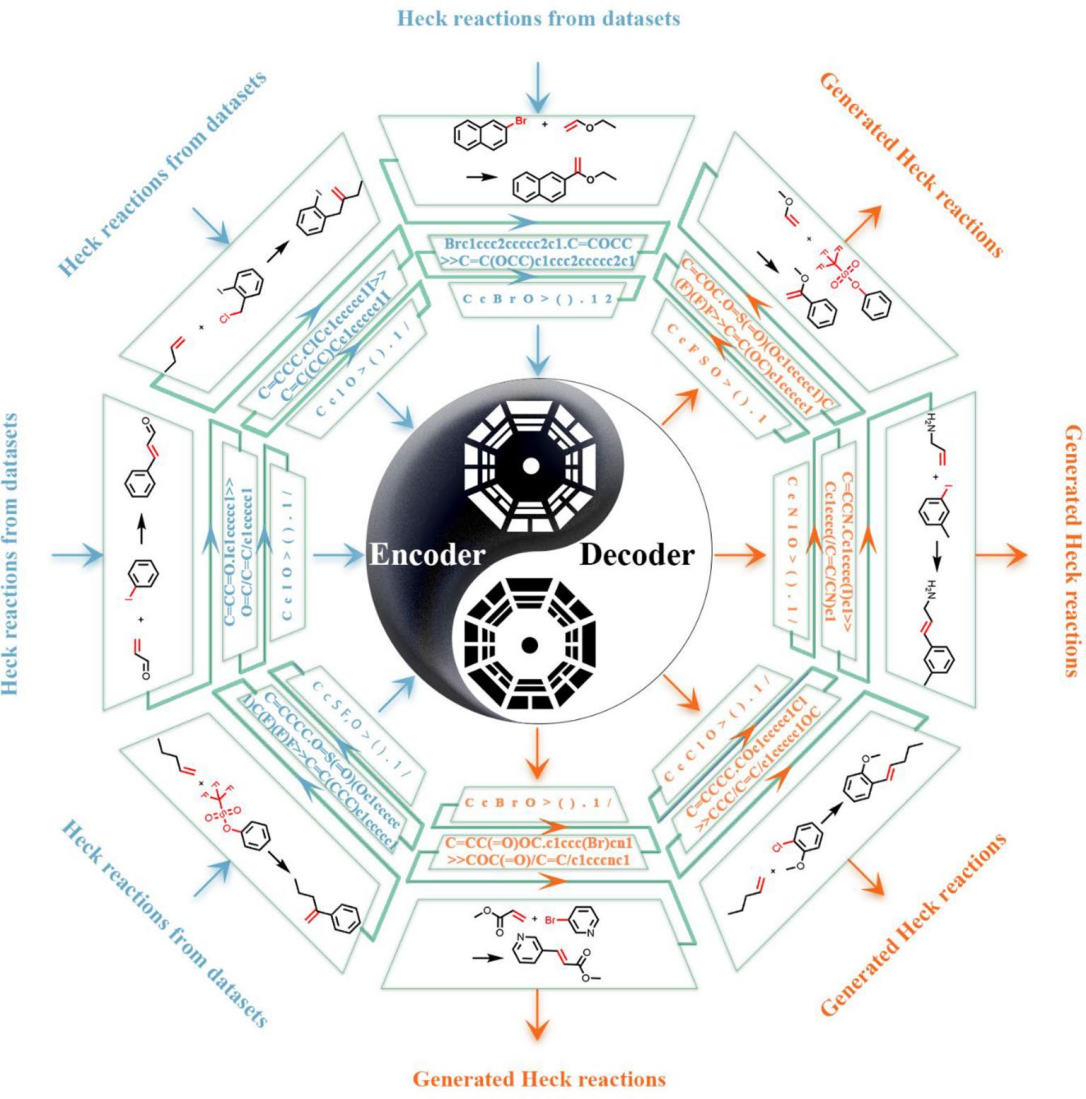
Schematic of the process for the generation of Heck coupling reactions. Heck reactions were imported into the encoder of the model after being converted from the 2-dimensional molecular graph to the 1-dimensional SMILES strings, then they were decoded into novel Heck reactions from the decoder

In this study, we applied the Transformer-XL model trained with Heck reaction for reaction generation (Fig. 1). We constructed a training dataset of the Heck coupling reactions. After reaction generating from the trained model, we organized 12 academic chemists to analyze and assess the thousands of generated reactions, which are not included in the training set. Then, we performed practical organic synthesis experiments to investigate the feasibility of the generated reactions as well as the accuracy of the configuration of the generated products. Based on the availability of raw materials, eight generated novel reactions were selected to. We attempted to investigate the proof-of-concept and feasibility of reaction generation using generative models represented by the Transformer model.

## Results and discussions

**Fig. 2:**
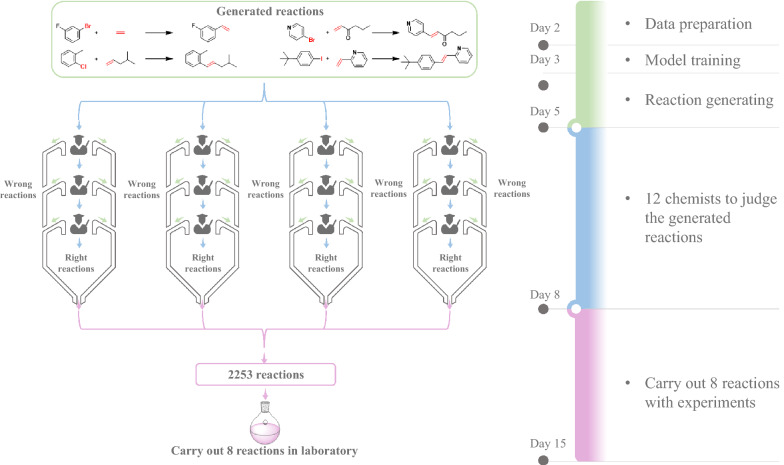
The flowchart of reaction generation and verification

**Fig. 3:**
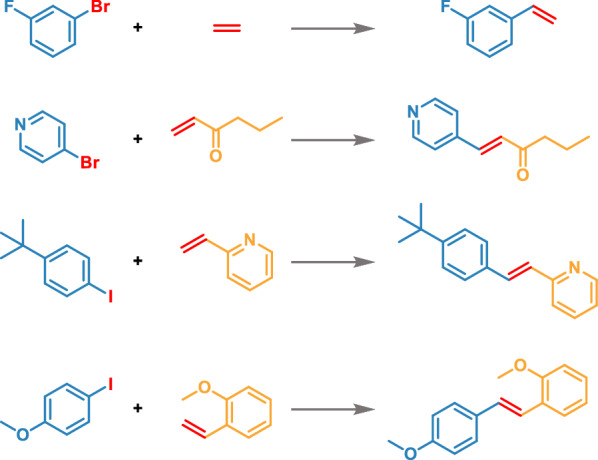
Examples of novel generated Heck reactions

We spent a total of 15 days exploring the novel Heck reactions using the Transformer-XL model (Fig. 2). First, we prepared the data (see “Method Dataset”) in 2 days, and then imported the training dataset comprising 8863 Heck reactions into the model. After 2 days of generating and then removing every duplicate, we obtained 4717 reactions not present in the training set. Then, 12 experimental chemists evaluated the validation of the reactions. The chemists were divided into four groups, and each group was responsible for a quarter of the total generated reactions. Only the reactions simultaneously considered as feasible by three chemists in the same group were retained and converged into to the final dataset, comprising 2253 reactions. A few representative examples of generated reactions are depicted in Fig. 3. These generated reactions are logical, with reasonable reactants and reaction centers matching Heck reactions. Furthermore, we spent 7 days to verify the reactions in a synthetic laboratory.

To evaluate the performance of our generative model, we applied the following metrics: validity, uniqueness, novelty and chemical feasibility (Table 1). For validity, we recorded the ratio of valid reactants, valid products and the fraction that all components in reaction are valid as 91.64%, 96.28% and 90.20%, respectively. Which indicates that our model captures explicit chemical constraints of molecular, such as proper valence, while generating reactions. We then compute the uniqueness of the reactions that all components are valid and the novelty which is the fraction of the unique reactions that are not present in training set. Chemical feasibility is the ratio of feasible reactions chosen by the chemists with the specialized knowledge and recorded as 47.76%.

**Table 1:** Performance metrics for generative model: validity, uniqueness, novelty and chemical feasibility

Reaction type	Validity			Uniqueness	Novelty	Chemical feasibility^a^
	Reactants	Products	All components			
Heck reaction	91.64%	96.28%	90.20%	15.03%	44.19%	47.76%

^a^The Chemical feasibility means the fraction of reactions that follow the Heck reactions' rule, such as stereoselectivity, in generated novel reactions

During the two-day process of generating reactions, we artificially assigned it as two steps, the generation of reactant and product molecules, and the process that corresponds the reactants to the products. It’s important to note that these two steps do not exist in the practical reaction generation, which is a continuous process that generated a complete reaction SMILES string from the “start of sequence”. We here divided it into two steps to help elaborate the concept of reaction generation. In stage I, the model generates reactants as well as products, which is like molecular generation. While in the process that corresponds the reactants to the products, which is the biggest difference between reaction generation and molecule generation, the products and reactants generated by the model must conform to the Heck reaction rules.

**Fig. 4:**
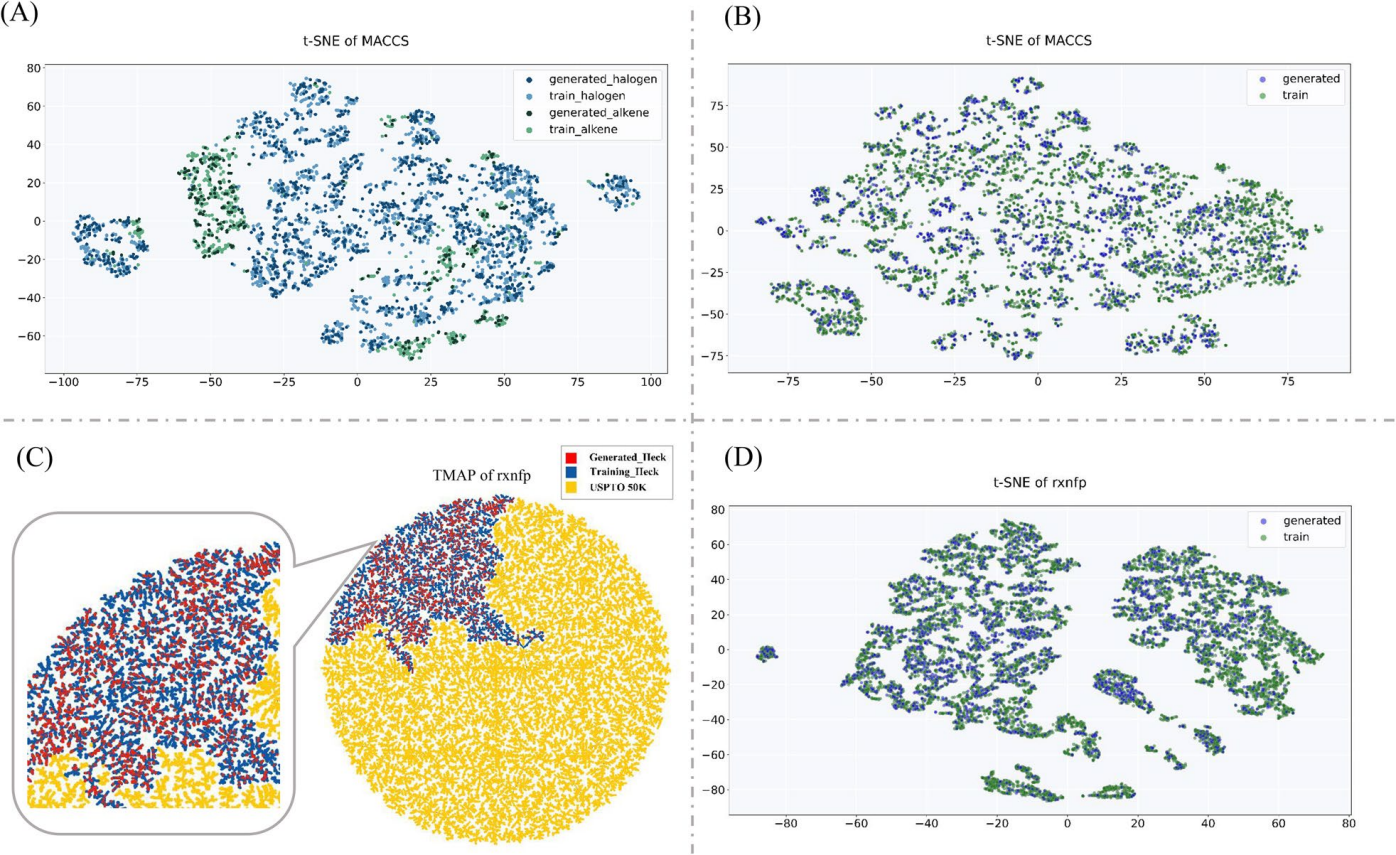
The plot of distribution of reactants, products and reactions. **A** The t-SNE plot of MACCS of reactants. Halogenated aromatics from the training set (blue) and generated set (deep blue), and alkenes from the training set (green) and generated set (deep green). **B** The t-SNE plot of products from the training set (green) and generated set (blue). **C** The TMAP plot of reactions from the training set (blue), generated set (red) and USPTO 50 K (yellow). **D** The t-SNE plot of *rxnfp* of reactions from the training set (green) and generated set (blue)

In stage I, each reaction is composed of at least one reactant and one product, so the prerequisite of a valid reaction is that both the reactant and product are effective. Notably, despite the 4717 novel reactions are not all valid reactions, all corresponding reactants and products were valid SMILES formulas, indicating that the molecules generative ability of the model is excellent. The t-distributed stochastic neighbor embedding (t-SNE) technique was used [30], which is similar to PCA, to visualize MACCS fingerprints to further verify the validation of generated molecules. The t-SNE approach is a variation of stochastic neighbor embedding [31] that visualizes high-dimensional data by providing each datapoint with a location in a two- or three-dimensional map. Moreover, MACCS is a molecular fingerprint with 166 dimensions, and each dimension corresponds to a functional group, suitable for reactants and products’ focus. Figure 4A shows the t-SNE plot of the MACCS fingerprints of the reactants of the generated reaction and those of the training set. The t-SNE plot of products is shown in Fig. 4B. As predicted, the training molecules entirely overlap with the corresponding generated molecular set, indicating that the model has generated numerous similar molecules around the training set.

Simultaneously, we classified the reactants in the training and generation sets including 2253 reactions. Table 2 lists the alkene distribution according to the number of substituents around the carbon–carbon double bond. Table 3 shows the classification of halogenated aromatic hydrocarbons and trifluoromethanesulfonate derivatives. It can be observed that the generated monosubstituted alkenes, bromo-aromatic hydrocarbons and iodinated aromatic hydrocarbons occupy the majority of the generated reactants; and this distribution is similar to that of these three reactants in the training set. Moreover, the generated reactions cover all alkene types. Although some types of alkenes are few in number, it remains indicative of the well-preserved integrity of molecular information in the process of molecular generation in stage I.

**Table 2:** Distribution of alkene reactants in the training set and generated set

Carbon–carbon double bond classification of reactant	Amount		Ratio (%)	
	Training	Generated	Training	Generated
Ethylene	141	9	1.57	0.40
Monosubstituted	8300	2179	92.60	96.72
Disubstituted	502	64	5.60	2.84
Trisubstituted	20	1	0.23	0.04
Total	8963	2253	100	100

**Table 3:** Distribution of halogenated aromatics and trifluoromethanesulfonate derivatives in the training set and generated set

Halogen atoms classification of reactants	Amount		Ratio (%)	
	Training	Generated	Training	Generated
Cl	471	161	5.3	7.1
Br	4939	1356	55.1	60.2
I	3274	670	36.5	29.8
OTf	279	66	3.1	2.9
Total	8963	2253	100	100

In stage II, the process of combining the corresponding reactant and product molecules into a reaction requires the model to learn the Heck reaction rules. Despite the Heck coupling reaction being among the most widely used catalytic carbon–carbon bond-forming tools in organic synthesis, the reactive rules are complex for the transformer-XL model. To confirm that the reactions generated by the model are exact Heck reactions, TMAP was used to visualize the reaction fingerprints (*rxnfp*) of the reactions. Schwaller et al*.* reported that the representations learned by the bidirectional encoder representations from transformers (BERT) can be used as *rxnfp*, which are independent of the number of molecules involved in a reaction [32]. Then, *rxnfp* were mapped to TMAP, a method used to visualize high-dimensional spaces as a tree-like graph [33]. As shown in Fig. 4C, we connected the 2253 chemist-judged reactions in the generated dataset to those in the training dataset according to the similarity measured by the *rxnfp*, each represented as a point. Additionally, the 50K reactions downloaded and curated by Liu et al*.* from the United States Patent Trademark Office (USPTO-50K) [19] were used to form the backbone of the chemical space, as it contains various chemical reactions. Color-coding the three classifications of reaction datasets above showed that the 2253 generated reactions and training set overlapped well, demonstrating that all the 2253 reactions judged by chemists are Heck coupling reactions. In addition, we verified the type of reaction using t-SNE to dimensionally reduce the *rxnfp* of the dataset (Fig. 4D). The result proved that the model has generated reactions that similar to the Heck reactions. And Additional file 1: Fig S11 shows the TMAP of training set, generated novel reaction set and USPTO 50K dataset. This is because the products of the removed reactions did not conform to the rule of Heck reactions, resulting in *rxnfp* of these reactions being quite different from the training set.

**Fig. 5:**
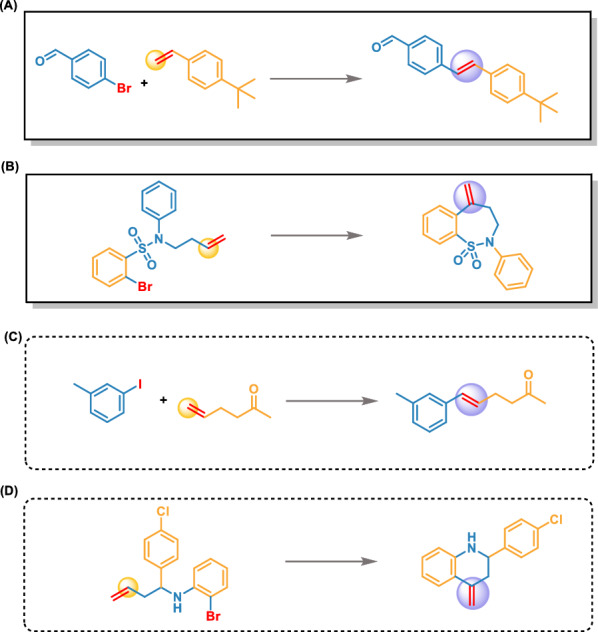
The representative examples of the intramolecular and intermolecular Heck reactions. **A** Intermolecular Heck reactions from the training set. **B** Intramolecular Heck reactions from the training set. **C** Intermolecular Heck reactions from the generated set. **D** Intramolecular Heck reactions from the generated set

To thoroughly investigate whether our model fully understands the Heck reaction, we conducted an in-depth analysis of the generated Heck reaction set. First, we divided the Heck reaction into intermolecular and intramolecular reactions. The training dataset contains 8464 intermolecular reactions and 499 intramolecular reactions (Table 4). The intermolecular reaction accounts for 98.2% of the generated reactions set, consistent with the characteristic of numerous intermolecular reactions present in the distribution of the training dataset. Several representative examples of intermolecular reactions and intramolecular reactions from the training and generated datasets are shown in Fig. 5.

**Table 4:** Distribution of Heck reactions in the training set and generated set

Classification of reaction type	Amount		Ratio (%)	
	Training	Generated	Training	Generated
Intermolecular reaction	8464	2213	94.4	98.2
Intramolecular reaction	499	40	5.6	1.8
Total	8963	2253	100	100

**Fig. 6:**
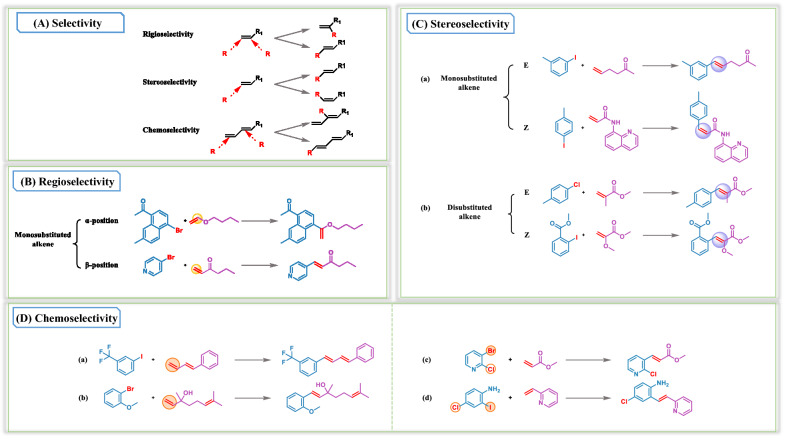
Analysis of the generated novel reactions from the perspective of the selectivity of the Heck reaction. **A** The definition of the regio-, stereo-, chemoselectivity. **B** Analysis of the regioselectivity of alkenes. **C** Analysis of the stereoselectivity of alkenes. **D** Analysis of the chemoselectivity of alkenes

The intermolecular reactions were analyzed with respect to the following three aspects: regioselectivity, stereoselectivity, and chemoselectivity. Based on the Heck reaction mechanism, the migration insertion of alkenes is the determining step of regioselectivity, whereas stereoselectivity involves the elimination of β hydrogen at the carbon–carbon double bond. Therefore, the regioselectivity and stereoselectivity of the generated reactions with respect to alkenes were analyzed. Regioselectivity indicates that there is one functional group that can react in two sites, and a reagent must select the reaction site (Fig. 6A). Regioselectivity has remained an unavoidable issue for Heck coupling reactions. For reactions with ethylene as the reactant, the occurrence of reactions does not involve regioselectivity, because the left and right alkene sites are equivalent for insertion. Moreover, disubstituted and trisubstituted alkenes are unfavorable for discussion with respect to regioselectivity. Therefore, we mainly discuss the regioselectivity of monosubstituted alkenes. Generally, the regioselectivity of monosubstituted alkenes is determined by the group attached to the double bond. As shown in Fig. 6B, 4-bromopyridine reacted with hex-1-en-3-one to produce 1,2-disubstituted alkenes, because of the formation of a new carbon–carbon bond at the opposite end of the alkene when the alkene is polarized by an electron-withdrawing group. The carbonyl group around the alkene in 1-hexanone is an electron withdrawing group, so the reaction site is located at the β position. Owing to steric hindrance, the arylation of monosubstituted alkenes is likely to occur at the β-position. Moreover, we observe that more reaction sites are located at β-positions in the generated reactions (Table 5). In contrast, electron-donating groups lead to form the 1,1-disubstituted product, such as the ether group. As another example shown in Fig. 6B, the model could use the information that the oxygen located in ethers is an electron-donating group and consequently produce a 1,1-disubstituted product.

**Table 5:** Regio- and stereoselectivity of monosubstituted alkenes in the training set and generated set

Classification of monosubstituted alkenes	Amount		Ratio (%)	
	Training	Generated	Training	Generated
α-position	440	62	5.59	2.90
β-position				
E	7397	2078	94.04	97.06
Z	29	1	0.37	0.04
Total	7866	2141	100	100

To further elucidate the understanding level of stereoselectivity of Heck reaction of our model, we have provided an introduction to stereoselectivity in Fig. 6A, referring to how they reacted (stereochemistry of the products). Ethylene and trisubstituted alkenes are not within the scope of this discussion because of the absence of existing stereoselectivity. For monosubstituted alkenes, stereoselectivity and regioselectivity are partially correlated. Only one case of stereoselectivity for monosubstituted alkenes exists if the reaction site is located at the α-position, similar to ethylene, where alkenes become terminal alkenes. However, when the reaction site is located at the β-position, the situation is complicated. As shown in Fig. 6C(a), when 1-iodo-3-methylbenzene reacts with hex-5-en-2-one, the product is an *E*-isomer, because the trans-alkene product, which is more stable on thermodynamics, is easily obtained. Only β-hydrogens located on the same side of the Pd atom can be eliminated and the steric hindrance of the substituent around the carbon–carbon double bond. In the generated reactions, we also observe (*Z*)-1,2 disubstituted alkenes in the product (Fig. 6C(a)). Though the most commonly observed products are (*E*)-1,2 disubstituted alkenes, extensive literature regarding (*Z*)-1,2 disubstituted alkenes were reported by Cheng et al*.*[34]. In terms of disubstituted alkenes, β-hydrogen elimination occurrs when the benzene ring is coplanar with the small substituent. The benzene ring and the large sterically hindering group are trans-coplanar when the product is generated. As illustrated in Fig. 6C(b), the benzene ring is coplanar with methyl or methoxy because of the steric hindrance of methoxycarbonyl; then, the *E*-isomer or *Z*-isomer product is generated. Table 5 lists the number of reactions in all categories of the stereo configuration of monosubstituted alkenes. It demonstrated that the model learned the rules that the amount of *E*-isomer products is significantly more than that of the *Z*-isomer products in the training set.

Finally, we discuss the degree to which the model learned the chemoselectivity of intermolecular reactions. Chemoselectivity is the preferential reactivity of one functional group over another (Fig. 6A). However, chemoselectivity is related to alkenes as well as halogenated aromatics. From Fig. 6D(a) and (b), it is obvious that the benzene ring preferentially reacts with the monosubstituted alkenes when monosubstituted double bonds, disubstituted or trisubstituted double bonds are simultaneously present in the reactants. Because the number of substituents at the carbon–carbon double bond determines the reactivity of the alkenes, the reaction rate and yield decrease with the increasing number of substituents. Among the four types of alkenes in our classification, the reactions with trisubstituted alkenes generally exhibit the lowest reaction rate and yield.

Similarly, in the presence of multiple halogens on the aromatic ring, alkenes prefer one of the halogens to react. As shown in Fig. 6D(c), although bromine and chlorine are both reactive sites, the model suggests that bromine preferentially reacts over chlorine. Similarly, the model suggests that the reaction activity of iodine is greater than that of chlorine, as shown in Fig. 6D(d). We further observe that the reaction priority is in the order of I >  > OTf > Br >  > Cl. This is owing to the different reaction rates of various halogenated aromatic hydrocarbons during the oxidative addition process of Heck reactions. Among them, iodoaromatics exhibit a high reaction rate as well as yield and only require mild reaction conditions, so they are the most commonly used Heck reaction substrates. Chemists have also favored the brominated aromatic hydrocarbons due to their inexpensiveness. Although trifluoromethanesulfonic acid derivatives exhibit high reactivities, they are rarely used because of the unavailability of raw materials which would lengthen the duration of the experiment. These aforementioned reasons also explain why the bromine and iodine reactions distinctly account for the majority in the training and generation sets listed in Table 4.

**Fig. 7:**
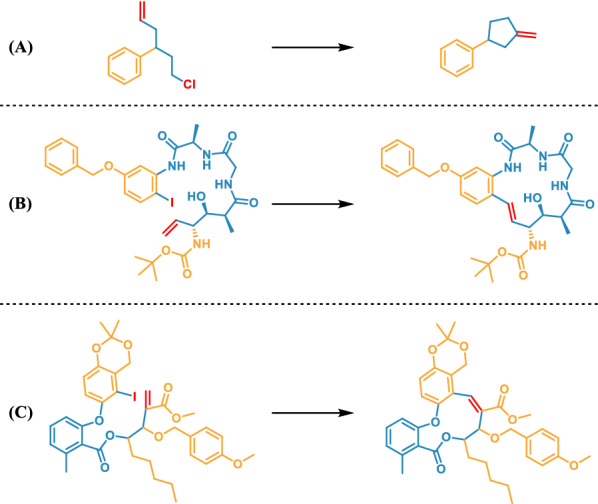
Examples of the generated intramolecular Heck reactions. **A** The intramolecular reaction that preferentially generate small cyclic compounds. **B** The intramolecular reaction that preferentially generate large cyclic compounds. **C** The intramolecular reaction that only generate endocyclized products

In the past three decades, intramolecular Heck reactions have emerged as a particularly versatile and reliable carbon–carbon bond-forming process, allowing for the formation of the whole spectrum of ring sizes: small (n = 3 or 4), normal (n = 5, 6, or 7), medium (n = 8–14) and large (n > 14). For intramolecular reactions with β-hydrogens available for elimination on both sides of alkenes, the general ring formation rule is to generate small cyclic compounds preferentially when the ring size is normal [35]. For example, in the selection of generating a five-membered ring or a six-membered ring, the five-membered ring is preferentially generated (Fig. 7A). The successful application of this reaction that generates extra-ring double bonds is of great significance, because the exocyclic double bond is a major limitation in synthesis. Intramolecular Heck reactions also enable the synthesis of exo or endo medium-sized and large rings, and the products are predominantly produced in the *E*-form configuration, because the ring tension is moderately low in large rings. Figure 7B shows the 15-endo cyclization products. The situation changes when there is no β-hydrogen can be eliminated at one side of the alkenes. Figure 7C confirms the presence of only one kind of 11-endo cyclization product when there is only one position for Pd atom insertion.

In the analysis of intramolecular reactions as well as the region-, stereo-, and chemoselectivity of intermolecular reactions, we confirmed that all of the 2253 reactions are theoretically feasible, thus demonstrating that the model displayed a sufficient understanding of the rules of chemical reactions. Meanwhile, the generated reaction set matched the training data in Tables 4 and 5, which demonstrated that our model has learned and reproduced some rules in the training set.

**Fig. 8:**
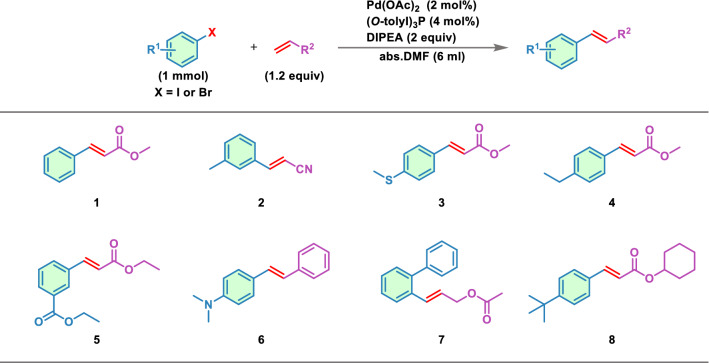
Pd(OAc)_2_-Catalyzed Heck reactions

Further, we conducted practical synthetic experiments to verify the feasibility of the reactions. We performed eight verification reactions based on the availability of the corresponding reactants and reagents. Table 6 shows the chosen reactions and the final products obtained in the laboratory. It showed that the products generated by the model and the real products obtained by the experiment are all in full compliance with our previous comparison analysis. Herein, we artificially chosen the reaction conditions for the verification reactions, because our model has insufficient understanding of reaction conditions, such as reaction temperature. We selected Pd(OAc)_2_, (O-tolyl)_3_P, and DIPEA as the catalyst, ligand, and base, respectively (Fig. 8). As a specific example, the generated product of reaction 1 shown in Table 6 are 1,2-disubstituted because of the electron-withdrawing group connected around the double bond. The expected ^1^H NMR and ^13^C NMR spectra of the products of the experiment with generated reactant (Additional file 1: Fig.S3). Similarly, the spectra of the other seven products are shown in Additional file 1: Fig. S4–S10. This demonstrated that the model accurately predicted the regioselectivity and stereoselectivity of these reactions.

**Table 6:**
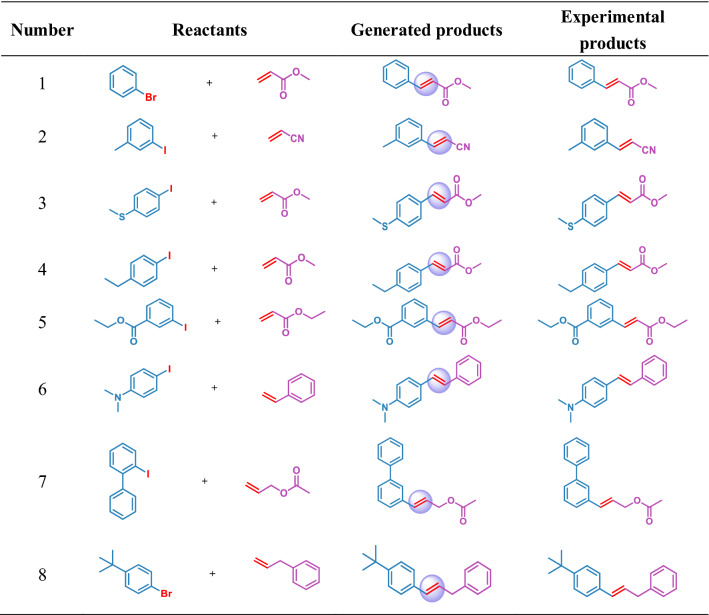
The comparison between generated reactions and experimental reactions

Moreover, there are also several types of errors observed in the generated reactions set, such as chirality error, carbon number error, heteroatom error, reaction type error, and chemoselectivity error. We recorded the frequency of these errors and listed them in Table 7. Among these errors, the reaction centers of all reactions but 14 reactions of the reaction-type errors are the Heck reaction center. That is, most reactions in the error set are caused by the neighbors of the reaction center. Moreover, the reaction center availability generated by the model is 98.42%. Notably, this differs significantly from the reaction prediction error. Although we discuss the reaction generation in two stages, the model generates reactions continuously and ceaselessly. It is challenging to generate reactions from de novo, because the model has to concurrently learn the reaction centers of the Heck reactions and the contextual correspondence of the SMILES sequence. However, our model still successfully learned and generated the Heck reaction center through training, indicating that it is successful in reaction generation. Additional file 1: Tables S1–S3 shows the distribution of the reactions with feasible reaction centers. And the distribution of these reactions also matched the training set.

**Table 7:**
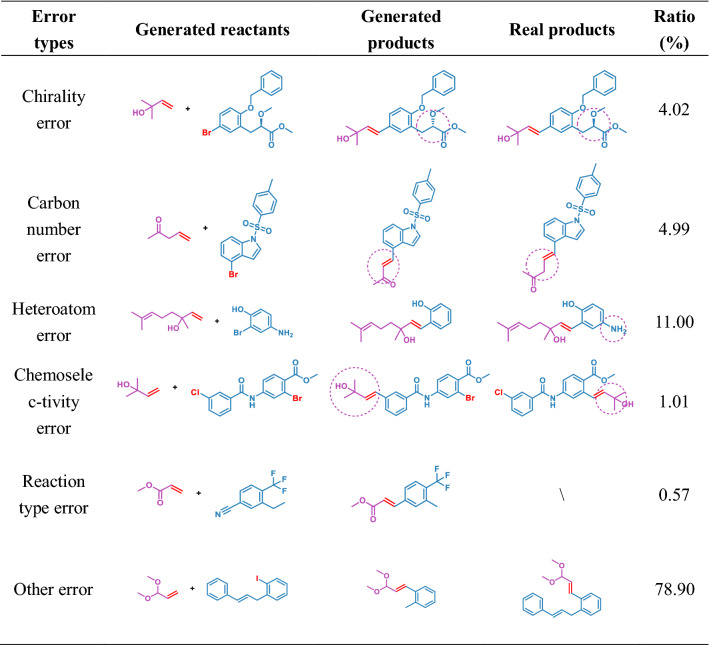
The classification of error types in generated reactions

Moreover, we tried to train the model with a larger set of reactions, Suzuki reactions, to evaluate the generative capacity of our model. The mechanism is shown in Additional file 1: Fig. S12. Table 8 shows the performance metric for our model trained with 78,032 Suzuki reactions.It is found that uniqueness, novelty and usability increased from 15.03%, 44.91% and 47.76% to 88.61%, 79.91% and 64.01%, respectively. And we trained the model with 8695 Kumada reactions (Table 8), The mechanism is shown in Additional file 1: Fig. S13.

**Table 8:** Performance metrics for the generative model trained with Suzuki reactions and Kumada reactions: validity, uniqueness, novelty and chemical feasibility

Reaction type	Validity			Uniqueness	Novelty	Chemical feasibility
	Reactants	Products	All components			
Suzuki reaction	88.93%	94.50%	85.70%	88.61%	79.91%	64.01%
Kumada reaction	93.52%	95.74%	92.71%	14.10%	50.59%	45.99%

**Fig. 9:**
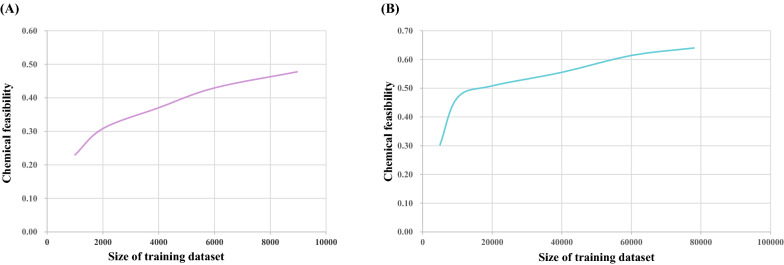
The influence of training set data size on chemical feasibility. **A** The relationship between chemical feasibility and the size of Heck reaction training dataset. **B** The relationship between chemical feasibility and the size of Suzuki reaction training dataset

We found that the chemical feasibility of the generated Heck reactions is 47.76%, while the chemical feasibility of Suzuki reactions is 64.01%, which is 16.25% higher. Therefore, we hypothesize that the chemical feasibility is related to the size of the training set, since the size of the training data determines the chemical space that can be explored. To verify our conjecture, we trained the model with different sizes of the Heck reaction and Suzuki reaction training datasets, and compared their chemical feasibility. As shown in Fig. 9, the chemical feasibility of Heck reactions and Suzuki reactions increased with the expanding of training set. Furthermore, the tendencies of chemical feasibility are still growing. This indicates that the main factor influencing chemical feasibility at the present stage is training dataset size. Although the chemical feasibility of generated Heck and Suzuki reactions currently are relatively low, their chemical feasibility would be higher with larger datasets.

Further, we compared our model with a simple recurrent VAE (RNN) and a recurrent VAE with an added attention layer (RNNAttn) [36]. The metrics were shown in Additional file 1: Table S7, though the uniqueness and novelty of the RNN and RNNAttn model are higher than our model, the validity and chemical feasibility of our model is more outstanding. Which indicates that our model has more sufficient understanding level of chemical reaction than others.

## Conclusion

In this manuscript, we trained the transformer-XL model with a dataset containing 8863 Heck reactions, and obtained 2253 novel Heck reactions evaluated by chemists. We further analyzed whether the model learned the rules of the Heck reaction based on evaluation of the regioselectivity, stereoselectivity, and chemoselectivity. Eight representative generated reactions were further verified by performing synthetic experiments, indicating that the consistency of the generated and experimental products. We demonstrated the feasibility of reaction generation of the transformer-XL model, which exhibited a thorough comprehension of the reactions, showing its ability to generate feasible and novel reactions.

It is challenging to quantitively measure the quality of the generation task used in natural language processing, such as poetry, novels, and molecular generation. However, the results obtained by our reaction generation task have only two unambiguous outcomes: right or wrong. Although further optimization is needed, it still provides new insights into the exploration of chemical reactions. We hope that the combination of AI and chemical reactions can provide helpful strategy in exploring novel chemical reactions.

## Methods

### Dataset

The reaction generative model is trained on a SMILES file containing only Heck coupling reactions, which are extracted from the “Reaxys” database based on the retrieval of reaction template and/or reaction name (all entries that use the phrase “Heck reaction”). The extracted Microsoft Excel files undergo a series of postprocessing processes with python scripts to obtain a high-quality dataset meeting the requirement of generating new reactions. In this step, inadequate reactions that the SMILES string is missing corresponding to either reactant or products and that have the same reactant and product are removed from the file. And for reactions with identical reaction SMILES we retained only one copy. Finally, a dataset containing 9959 Heck reaction is connected based on Heck reaction template with a Python script utilizing the RDkit and is divided into training set and validation set (9:1).

### Model

We selected the Transformer-XL model as the generation model, which is a state-of-art method combined AI with the chemical field. It consists of encoder and decoder architecture, and an “attention” mechanism was added to connect the encoder and decoder. Because of the entire dependence on the attention mechanism, the model avoids recurrence and draws global dependencies between input and output. In addition, every encoder and decoder structure includes several feed-forward layers, in which the chemical information the Transformer-XL model learned from the training dataset stores. We first de-bugged the model before generating the reactions with the Transformer-XL model. Therefore, we build a series of explorations based on training Heck reaction dataset to effectively select hyperparameters, and the results are shown in Additional file 1: Tabless S4–S6.

**Fig. 10:**
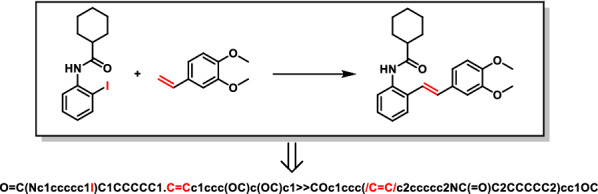
Mutual conversions between the SMILES language and the molecular structure

To match the algorithms of the Transformer-XL models. We imported the reactions with “simple molecular-input line-entry system” (SMILES) strings. We use letters to represent atoms and numbers to represent the number of rings. For example, in Fig. 10, we apply c1ccccc1 to present benzene, character “ >  > ” to separate reactants and products, “.” to separate different reactants. Before the training step, the model will construct a vocab (v1, …, vi) that contains all characters in the SMILES strings.

**Fig. 11:**
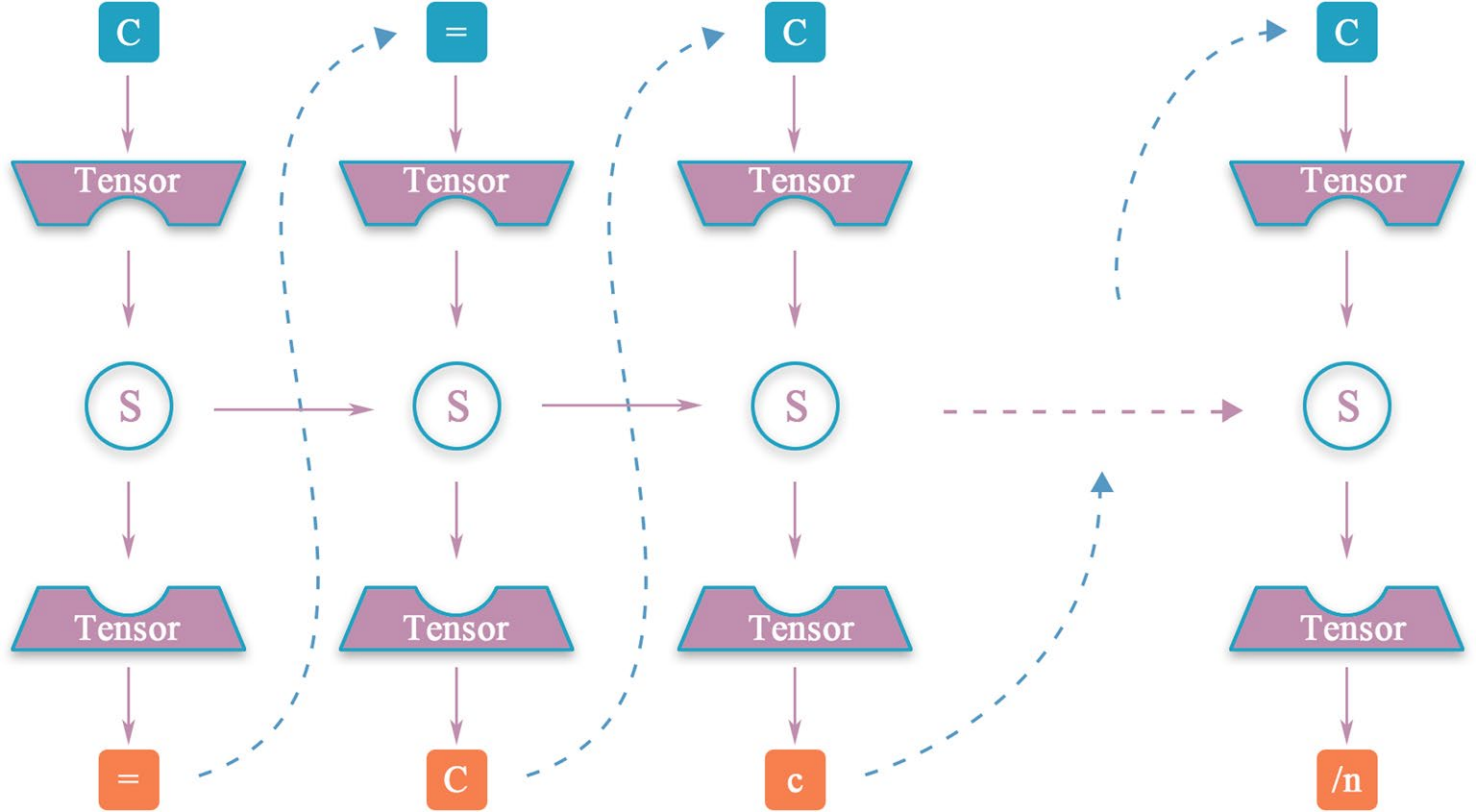
The general flow of reaction formation. We randomly selected a symbol C to start the generation, a tensor of each character of vocab then imports into the model and the probability distribution P as an outcome. According to the distribution P, the model randomly selected the next symbol. Above steps will be cyclically execute until the appearance of EOF. Finally, the SMILES string as the result to output

The inferenced part learns from the inference of poems [37]. This part inference the whole reaction with a start symbol. The model gives a tensor T (t1, …, ti) based on the start symbol and the data from the training step. The model then outcome the probability distribution P (s1, …, si) of the next symbol. The distribution P is estimated based on the tensor T and the built-in functions softmax in the tensorflow, which is defined as

1 $$P({s}_{k})= \frac{\mathrm{exp}({t}_{k})}{{\sum }_{{k}^{^{\prime}}=1}^{i}\mathrm{exp}({t}_{{k}^{^{\prime}}})}$$

where t_k_ corresponds to the kth element of tensor T. Then the model randomly selects the next symbol according to the probability P, and feedback to the model to find the following symbol (see Fig. 11). To indicate the SMILES string happens to be a reaction, we lead the character “\n” into each reaction SMILES as “end of line” (EOF). So the model will outcome the result and restart the generation of SMILES string from scratch when it detects the formation of EOF.

## Supplementary Information

**Additional file 1: Figure S1. **General mechanism of Heck coupling reaction. **Figure S2.** The conditions of preparation of compounds 1-8. **Figure S3. **^1^H NMR and ^13^C NMR spectra of methyl cinnamate (1). **Figure S4.** ^1^H NMR and ^13^C NMR spectra of (*E*)-3-(m-tolyl)acrylonitrile (2). **Figure S5. **^1^H NMR and ^13^C NMR spectra of methyl (*E*)-3-(4-(methylthio)phenyl)acrylate (3). **Figure S6. **^1^H NMR and ^13^C NMR spectra of methyl (*E*)-3-(4-ethylphenyl)acrylate (4). **Figure S7. **^1^H NMR and ^13^C NMR spectra of ethyl (*E*)-3-(3-ethoxy-3-oxoprop-1-en-1-yl)benzoate (5). **Figure S8. **^1^H NMR and ^13^C NMR spectra of (*E*)-N,N-dimethyl-4-styrylaniline (6). **Figure S9. **^1^H NMR and ^13^C NMR spectra of (*E*)-3-([1,1'-biphenyl]-2-yl)allyl acetate (7). **Figure S10. **^1^H NMR and ^13^C NMR spectra of cyclohexyl (*E*)-3-(4-(tert-butyl)phenyl)acrylate (8). **Table S1.** Distribution of the reactions that don’t have chemical feasibility in the generated set. **Table S2.** Distribution of alkene reactants of the reactions that don’t have chemical feasibility in the generated set. **Table S3.** Distribution of halogenated aromatics and trifluoromethanesulfonate derivatives of the reactions that don’t have chemical feasibility in the generated set. **Figure S11. **The TMAP plot of reactions from training set (blue), generated novel reaction set (red) and USPTO 50K (yellow). **Figure S12.** General mechanism of Suzuki reaction. **Figure S13.** General mechanism of Kumada reaction. **Table S4.** The validity of the Transformer-XL model with different batch sizes. All are trained on a 1080 GPU and hidden_size = 512, drop_out = 0.1, n_head = 8, layer = 12. **Table S5.** The validity of the Transformer-XL model with different hidden sizes. All are trained on a 1080 GPU and batch_size = 64, drop_out = 0.1, n_head = 8, layer = 12. **Table S6.** The validity of the Transformer-XL model with different drop out. All are trained on a 1080 GPU and batch_size = 64, hidden_size = 512, n_head = 8, layer = 12. **Table S7.** Performance metrics for the different generative models: validity, uniqueness, novelty and availability.

## Data Availability

The model and processed data sets will be made available at https://github.com/hongliangduan/From-Theory-to-Experiment-Transformer-Based-Generation-Enables-Rapid-Discovery-of-Novel-Reactions.git.
