# Supplementary material for: From theory to experiment: transformer-based generation enables rapid discovery of novel reactions
Source: J Cheminform. 2022 Sep 2;14:60. doi: 10.1186/s13321-022-00638-z (PMC9438336; doi:10.1186/s13321-022-00638-z)
Supplement: Supplementary file 1 — Additional file 1: Figure S1. General mechanism of Heck coupling reaction. Figure S2. The conditions of preparation of compounds 1-8. Figure S3. 1H NMR and 13C NMR spectra of methyl cinnamate (1). Figure S4. 1H NMR and 13C NMR spectra of (E)-3-(m-tolyl)acrylonitrile (2). Figure S5. 1H NMR and 13C NMR spectra of methyl (E)-3-(4-(methylthio)phenyl)acrylate (3). Figure S6. 1H NMR and 13C NMR spectra of methyl (E)-3-(4-ethylphenyl)acrylate (4). Figure S7. 1H NMR and 13C NMR spectra of ethyl (E)-3-(3-ethoxy-3-oxoprop-1-en-1-yl)benzoate (5). Figure S8. 1H NMR and 13C NMR spectra of (E)-N,N-dimethyl-4-styrylaniline (6). Figure S9. 1H NMR and 13C NMR spectra of (E)-3-([1,1'-biphenyl]-2-yl)allyl acetate (7). Figure S10. 1H NMR and 13C NMR spectra of cyclohexyl (E)-3-(4-(tert-butyl)phenyl)acrylate (8). Table S1. Distribution of the reactions that don’t have chemical feasibility in the generated set. Table S2. Distribution of alkene reactants of the reactions that don’t have chemical feasibility in the generated set. Table S3. Distribution of halogenated aromatics and trifluoromethanesulfonate derivatives of the reactions that don’t have chemical feasibility in the generated set. Figure S11. The TMAP plot of reactions from training set (blue), generated novel reaction set (red) and USPTO 50K (yellow). Figure S12. General mechanism of Suzuki reaction. Figure S13. General mechanism of Kumada reaction. Table S4. The validity of the Transformer-XL model with different batch sizes. All are trained on a 1080 GPU and hidden_size = 512, drop_out = 0.1, n_head = 8, layer = 12. Table S5. The validity of the Transformer-XL model with different hidden sizes. All are trained on a 1080 GPU and batch_size = 64, drop_out = 0.1, n_head = 8, layer = 12. Table S6. The validity of the Transformer-XL model with different drop out. All are trained on a 1080 GPU and batch_size = 64, hidden_size = 512, n_head = 8, layer = 12. Table S7. Performance metrics for the different generative models: validity [file 13321_2022_638_MOESM1_ESM.docx]

**From theory to experiment: transformer-based generation enables rapid discovery of novel reactions**

Xinqiao Wang^1†^, Chuansheng Yao^3, 4†^, Yun Zhang^1†^, Jiahui Yu^1^, Haoran Qiao^5^, Chengyun Zhang^1^, Yejian Wu^1^, Renren Bai^3,^ ^4*^ & Hongliang Duan^1,2*^

^1^Artificial Intelligence Aided Drug Discovery Institute, College of Pharmaceutical Sciences, Zhejiang University of Technology, Hangzhou 310014, P. R. China

^2^State Key Laboratory of Drug Research, Shanghai Institute of Materia Medica (SIMM), Chinese Academy of Sciences, Shanghai 201203, China.

^3^College of Pharmacy, School of Medicine, Hangzhou Normal University, Hangzhou, PR China

^4^Key Laboratory of Elemene Class Anti-Cancer Chinese Medicines, Engineering Laboratory of Development and Application of Traditional Chinese Medicines, Collaborative Innovation Center of Traditional Chinese Medicines of Zhejiang Province, Hangzhou Normal University, Hangzhou, PR China

^5^College of Mathematics and Physics, Shanghai University of Electric Power, Shanghai, 201203 (P. R. China)

^†^Xinqiao Wang, Chuansheng Yao and Yun Zhang contributed equally to this work

*Corresponding Author: Hongliang Duan

Email: [hduan@zjut.edu.cn](mailto:hduan@zjut.edu.cn)

Renren Bai

Email: [renrenbai@hznu.edu.cn](mailto:renrenbai@hznu.edu.cn)

**Table of content**

[Section S1 General mechanism of Heck reaction 3](#_Toc47688573)

[Section S2 Experiment section 3](#_Toc47688574)

[S2.1 General information 3](#_Toc47688576)

[S2.2 General procedure for the preparation of compounds 1-8 4](#_Toc47688577)

[S2.3 Spectra of compounds 1-8 4](#_Toc47688578)

[Section S3 Table S1 to S3 13](#_Toc47688574)

[Section S4 Fig. S11 to S13 14](#_Toc47688574)

[Section S5 Exploration of hyperparameters 14](#_Toc47688574)

[Section S6 References 15](#_Toc47688580)

# Section S1 General mechanism of Heck reaction

We summarized the general mechanism of the Heck reaction in Fig. S1. The precursor of Pd(II) is first activated to form a low coordination number Pd(0) before the oxidative addition of palladium and halogenated hydrocarbon, and palladium is subsequently inserted into the halogen-carbon bond in this process [1-3]. The palladium atoms then interact with the alkene to produce π complexes, and the coordinated alkene is inserted migration into the palladium-carbon bond. Then β-H is eliminated after rotational isomerization into trans isomers with lower torsional tension, and a new intermediate with coordination between palladium and alkene is obtained. The alkenes and the Pd(II) species are also produced in the following process, and the Pd(II) was converted to Pd(0) by reduction and elimination under the effect of the base. Numerous chemists have devoted to Heck reaction research because of its influence, demonstrating that the Heck reaction quantities is sufficient to train the model [4].

**Fig. S1** General mechanism of Heck coupling reaction.

# Section S2 Experiment section

## S2.1 General information

All the starting materials were obtained from commercial sources and used without further purification. All the reactions were carried out under a dry nitrogen atmosphere. The 1H NMR and 13C NMR spectra were recorded on Bruker and Varian instruments at 500, 400 and 125, 100 MHz, respectively, where CDCl_3_ or DMSO-*d*_6_ was used as the solvent. *J* (coupling constants) is expressed in Hertz (Hz).

## S2.2 General procedure for the preparation of compounds 1-8

To a solution of halobenzene or substituted halobenzene (1 mmol) in DMF (6 mL) under an atmosphere of dry nitrogen, Pd(OAc)_2_ (2 mol%), (*O*-tolyl)_3_P (4 mol%), substituted olefin (1.2 equiv), and DIPEA (2 equiv) were added. The reaction solution was deoxidized by nitrogen displacement three times and then heated to 120 ^o^C for 12 h. The mixture was then naturally cooled to room temperature. 15 mL of water was added and the mixture was extracted three times with EtOAc (3 × 15 mL). The combined organic layer was washed three times with saturated brine (3 × 15 mL), dried over anhydrous Na_2_SO_4_. The organic solvent was then removed under reduced pressure and separated by thin-layer silica gel (Fig. S2).

**Fig. S2** The conditions of preparation of compounds 1-8.

## S2.3 Spectra of compounds 1-8

Methyl cinnamate (1)


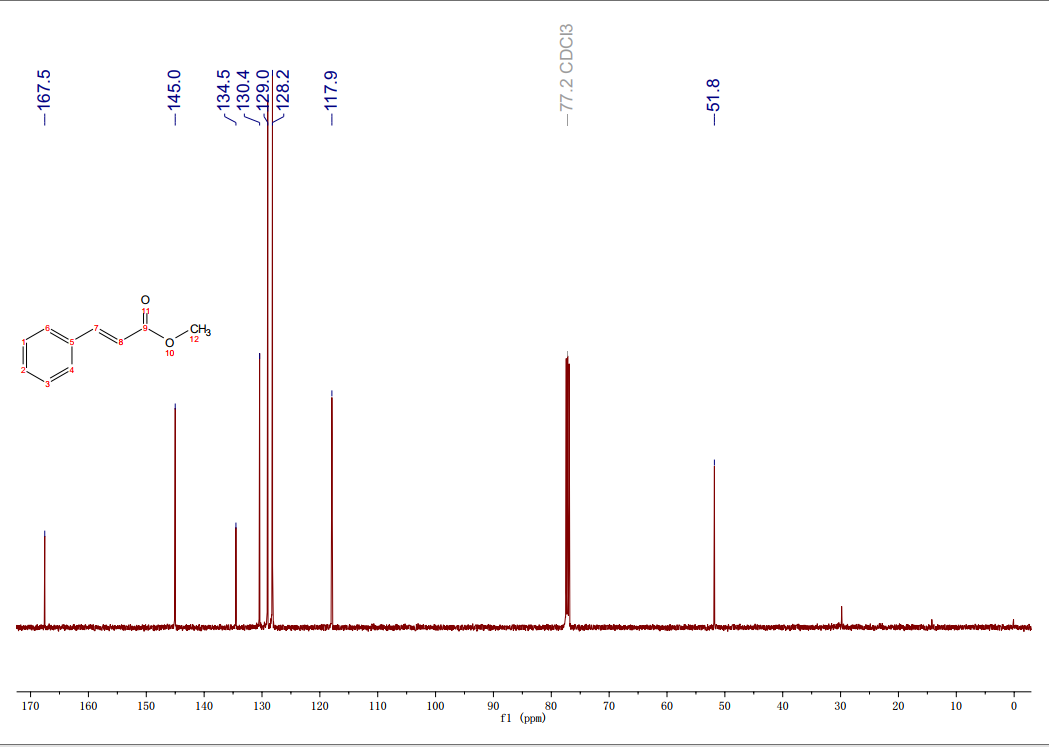

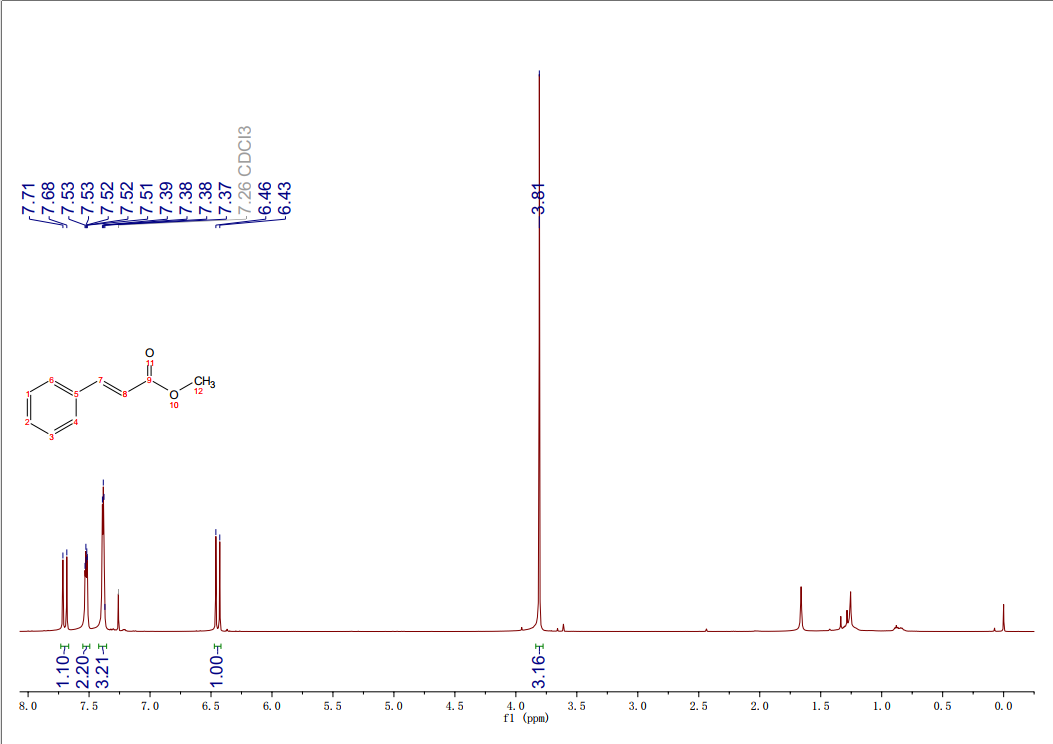
Yellowish solid, yield 61.7%; ^1^H NMR (500 MHz, CDCl_3_) δ 7.70 (d, *J* = 15.0 Hz, 1H), 7.53 - 7.51 (m, 2H), 7.39 - 7.37 (m, 3H), 6.44 (d, *J* = 15.0 Hz, 1H), 3.81 (s, 3H); ^13^C NMR (125 MHz, CDCl3) δ 167.5, 145.0, 134.5, 130.4, 129.0, 128.2, 117.9, 51.8. ^1^H NMR and ^13^C NMR spectra of methyl cinnamate were shown in Fig. S3.

**Fig. S3** ^1^H NMR and ^13^C NMR spectra of methyl cinnamate (1).

(*E*)-3-(m-tolyl)acrylonitrile (2)


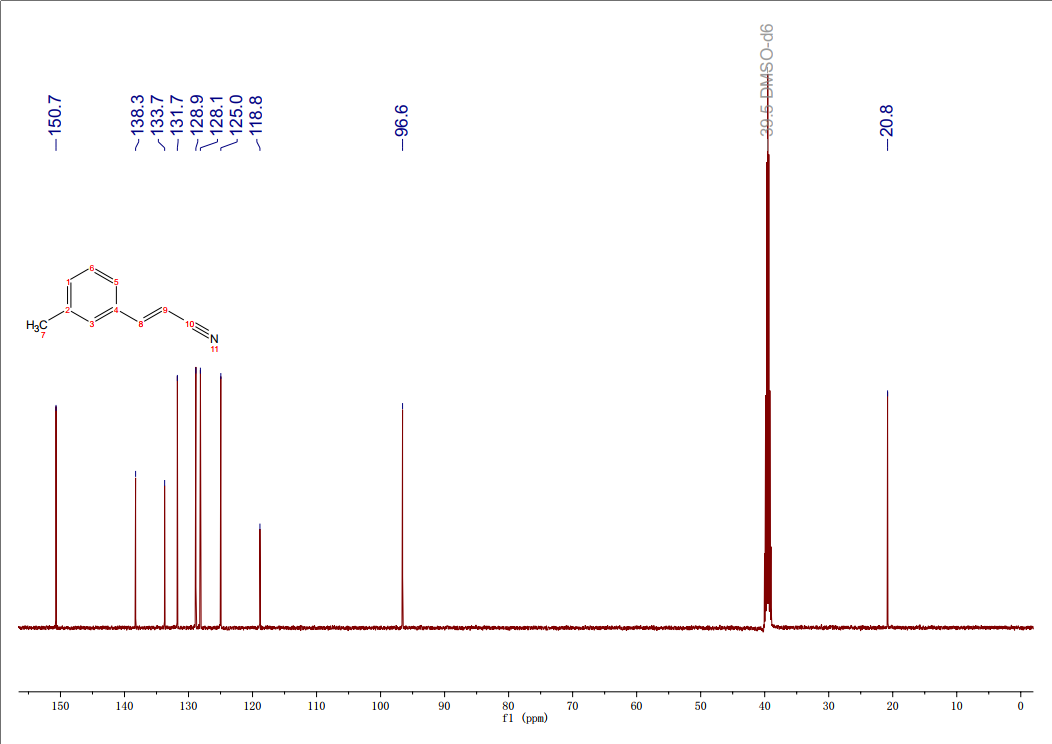

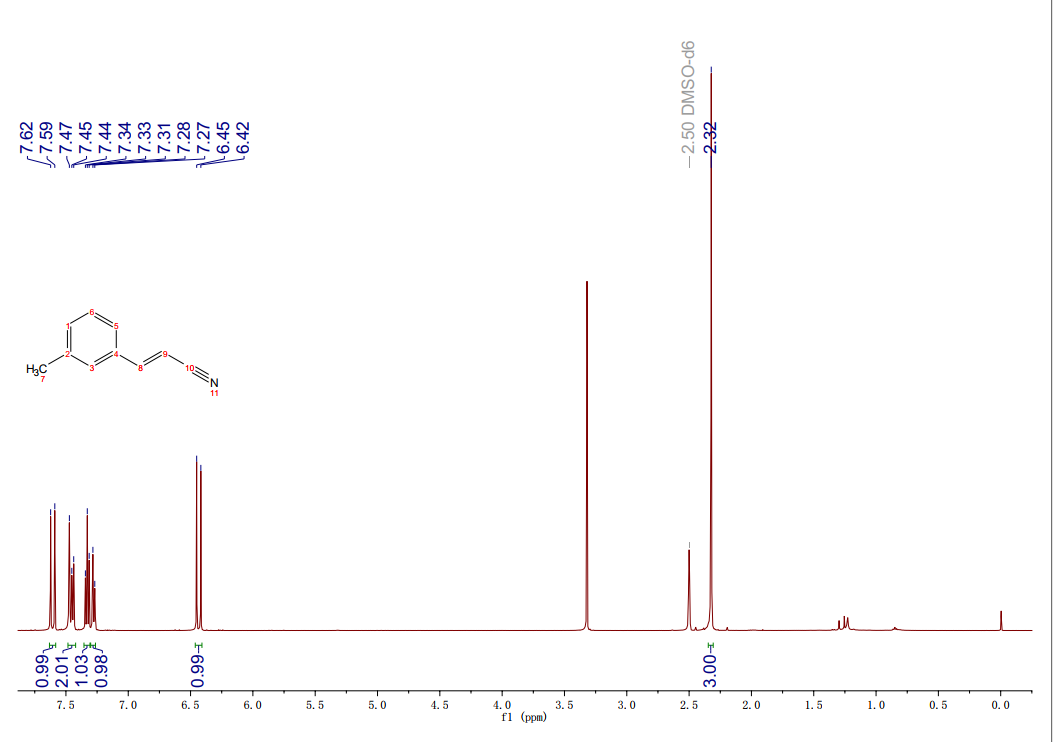
Colorless oil, yield 41.2%; ^1^H NMR (500 MHz, DMSO-*d*_6_) δ 7.61 (d, *J* = 15.0 Hz, 1H), 7.47 - 7.44 (m, 2H), 7.33 (t, *J* = 7.6 Hz, 1H), 7.28 (d, *J* = 5.0 Hz, 1H), 6.43 (d, *J* = 15.0 Hz, 1H), 2.32 (s, 3H); ^13^C NMR (125 MHz, DMSO-*d*_6_) δ 150.7, 138.3, 133.7, 131.7, 128.9, 128.1, 125.0, 118.8, 96.6, 20.8. ^1^H NMR and ^13^C NMR spectra of (*E*)-3-(m-tolyl)acrylonitrile were shown in Fig. S4.

**Fig. S4** ^1^H NMR and ^13^C NMR spectra of (*E*)-3-(m-tolyl)acrylonitrile (2).

Methyl (*E*)-3-(4-(methylthio)phenyl)acrylate (3)


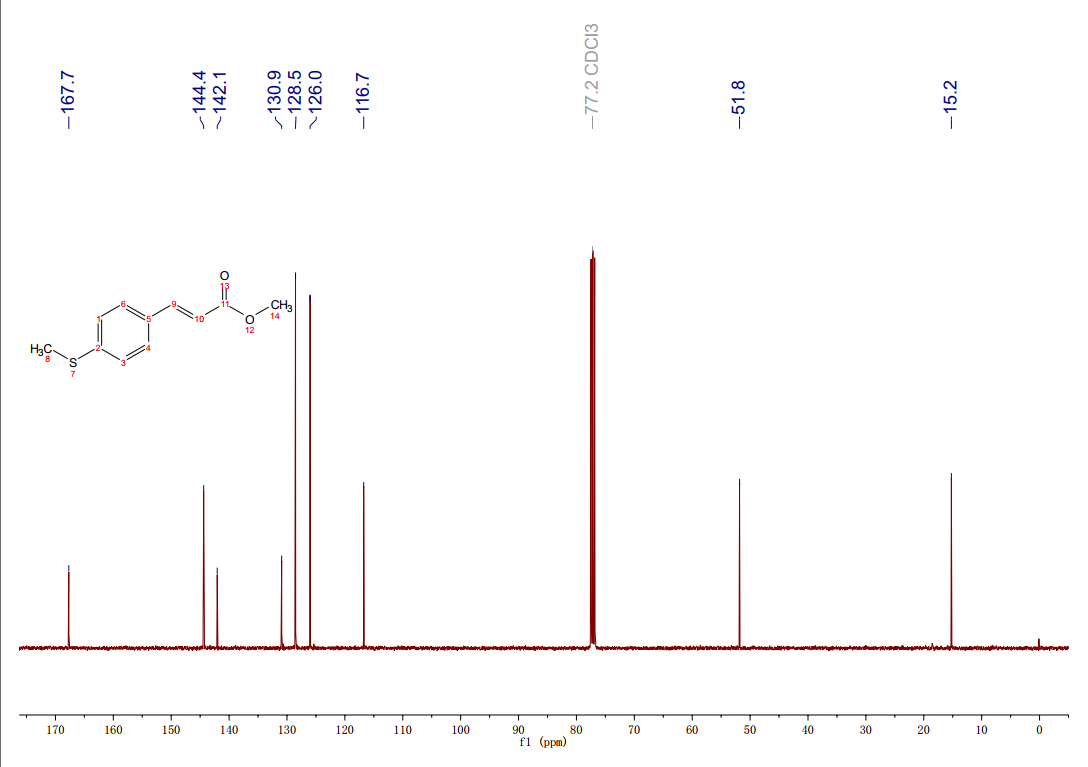

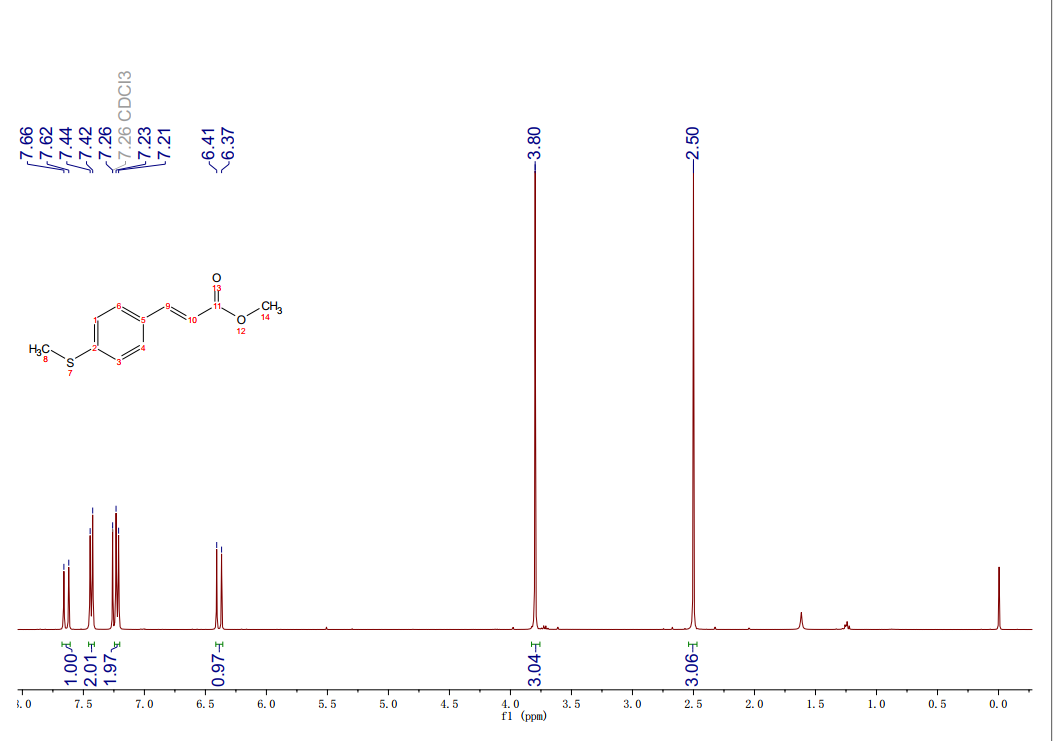
White solid, yield 54.8%; ^1^H NMR (400 MHz, CDCl_3_) δ 7.64 (d, *J* = 16.0 Hz, 1H), 7.43 (d, *J* = 8.0 Hz, 2H), 7.22 (d, *J* = 8.0 Hz, 2H), 6.39 (d, *J* = 16.0 Hz, 1H), 3.80 (s, 3H), 2.50 (s, 3H); ^13^C NMR (100 MHz, CDCl_3_) δ 167.7, 144.4, 142.1, 130.9, 128.5, 126.0, 116.7, 51.8, 15.2. ^1^H NMR and ^13^C NMR spectra of methyl (*E*)-3-(4-(methylthio)phenyl)acrylate were shown in Fig. S5.

**Fig. S5** ^1^H NMR and ^13^C NMR spectra of methyl (*E*)-3-(4-(methylthio)phenyl)acrylate (3).

Methyl (E)-3-(4-ethylphenyl)acrylate (4)


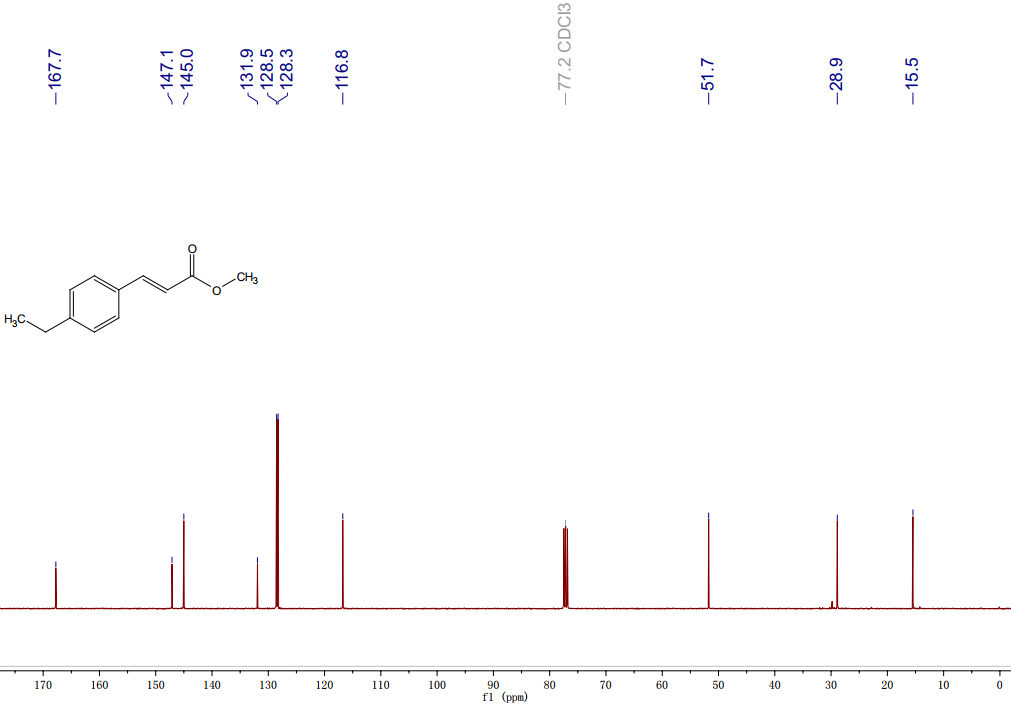

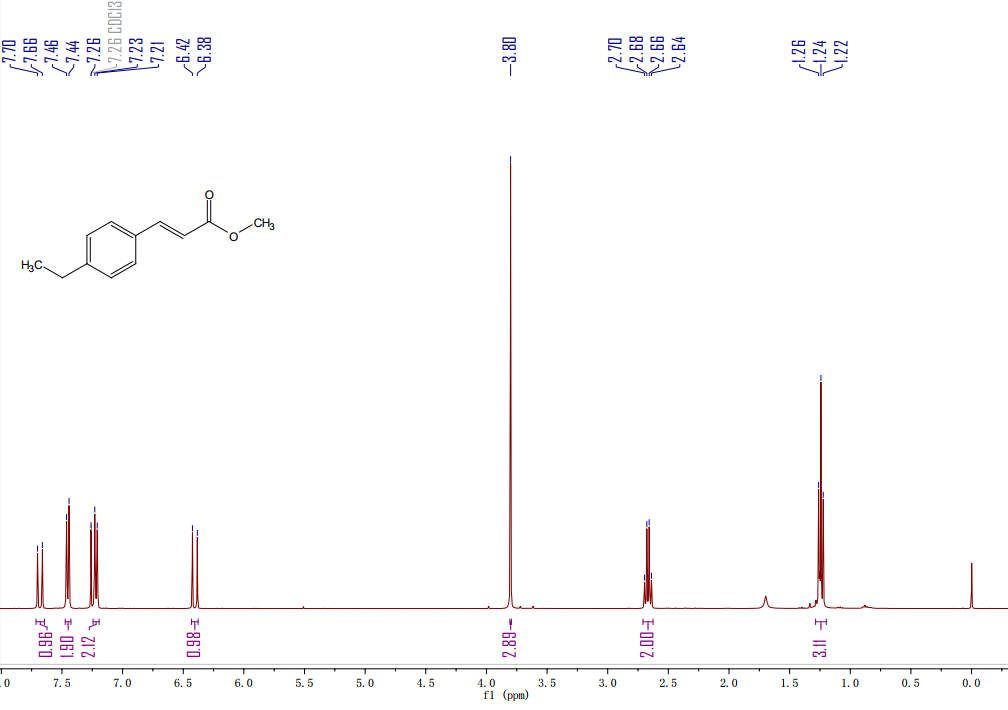
Colorless oil, yield 51.5%; ^1^H NMR (400 MHz, CDCl_3_) δ 7.68 (d, *J* = 16.0 Hz, 1H), 7.45 (d, *J* = 8.0 Hz, 2H), 7.22 (d, *J* = 8.0 Hz, 2H), 6.40 (d, *J* = 16.0 Hz, 1H), 3.80 (s, 3H), 2.67 (q, *J* = 7.6 Hz, 2H), 1.24 (t, *J* = 7.6 Hz, 3H); ^13^C NMR (100 MHz, CDCl_3_) δ 167.7, 147.1, 145.0, 131.9, 128.5, 128.3, 116.8, 51.7, 28.9, 15.5. ^1^H NMR and ^13^C NMR spectra of methyl (E)-3-(4-ethylphenyl)acrylate were shown in Fig. S6.

**Fig. S6** ^1^H NMR and ^13^C NMR spectra of methyl (*E*)-3-(4-ethylphenyl)acrylate (4).

Ethyl (*E*)-3-(3-ethoxy-3-oxoprop-1-en-1-yl)benzoate (5)


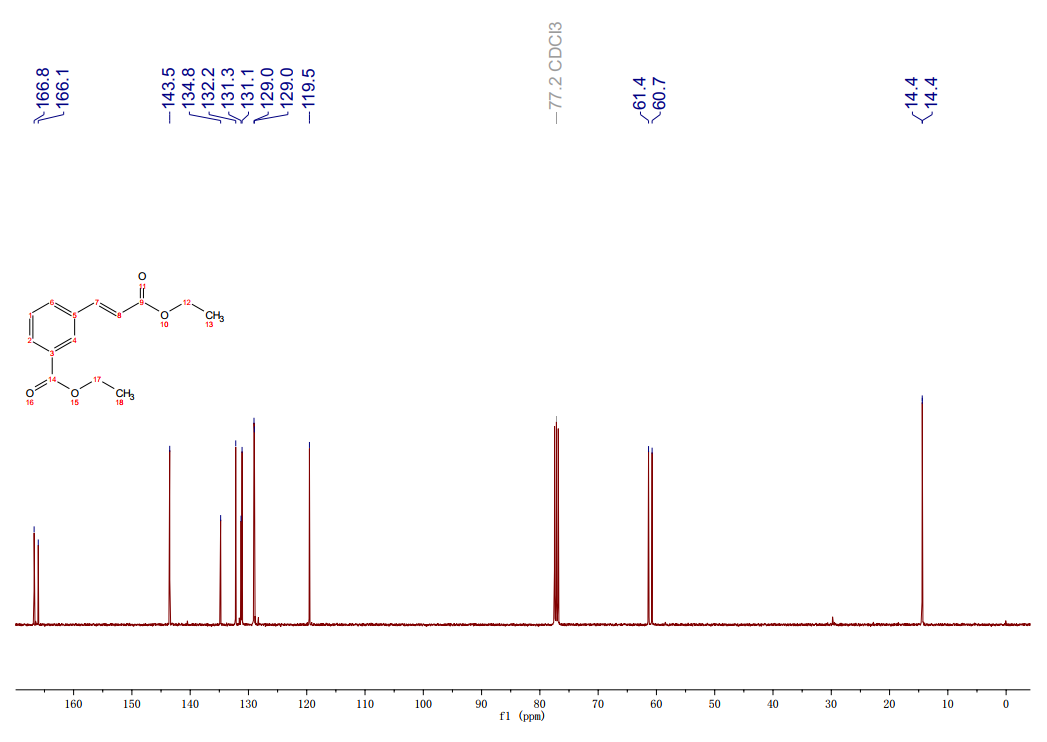

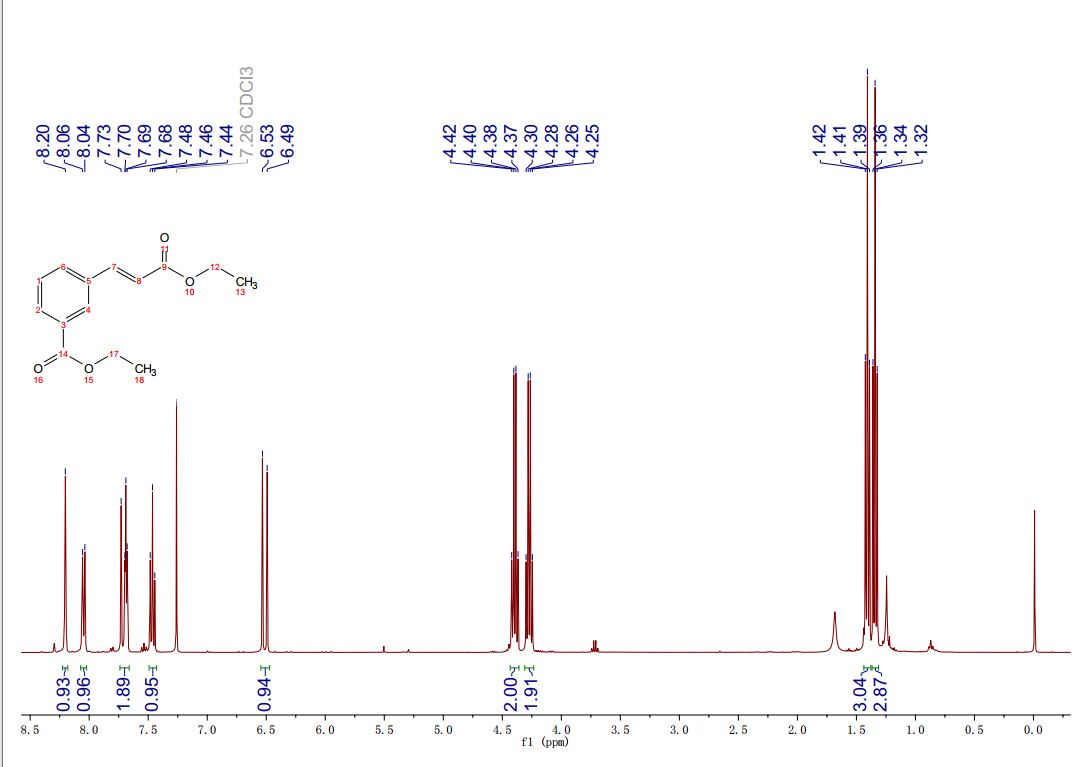
Colorless oil, yield 47.5%; ^1^H NMR (400 MHz, CDCl_3_) δ 8.20 (d, *J* = 1.7 Hz, 1H), 8.05 (d, *J* = 7.8, 1.4 Hz, 1H), 7.73 - 7.68 (m, 2H), 7.46 (t, *J* = 7.8 Hz, 1H), 6.51 (d, *J* = 16.0 Hz, 1H), 4.39 (q, *J* = 7.1 Hz, 2H), 4.27 (q, *J* = 7.1 Hz, 2H), 1.41 (t, *J* = 7.1 Hz, 3H), 1.34 (t, *J* = 7.1 Hz, 3H); ^13^C NMR (100 MHz, CDCl_3_) δ 166.8, 166.1, 143.5, 134.8, 132.2, 131.3, 131.1, 129.0, 129.0, 119.5, 61.4, 60.7, 14.4, 14.4. ^1^H NMR and ^13^C NMR spectra of ethyl (*E*)-3-(3-ethoxy-3-oxoprop-1-en-1-yl)benzoate were shown in Fig. S7.

**Fig. S7** ^1^H NMR and ^13^C NMR spectra of ethyl (*E*)-3-(3-ethoxy-3-oxoprop-1-en-1-yl)benzoate (5).

(*E*)-N,N-dimethyl-4-styrylaniline (6)


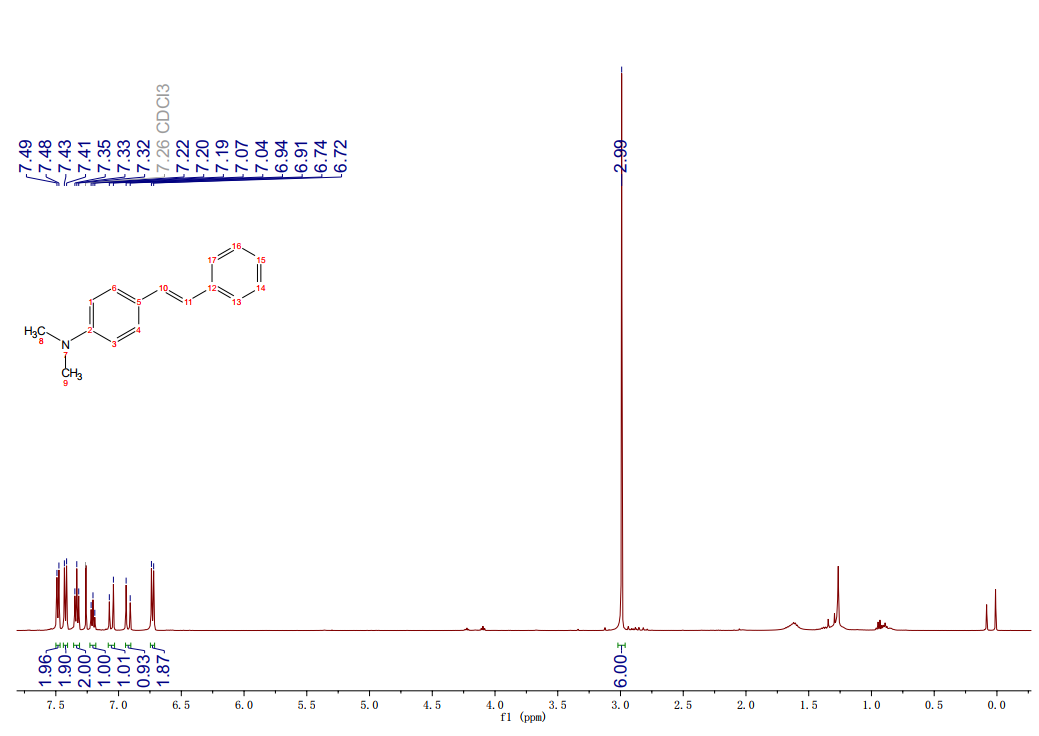

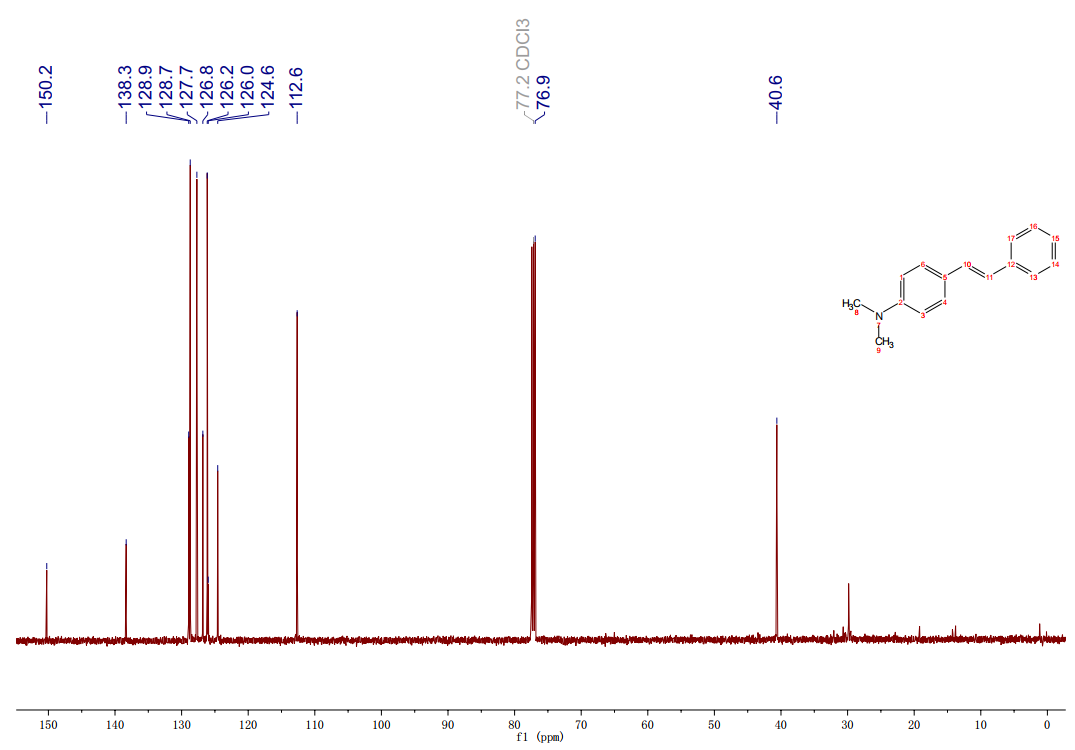
Yellowish solid, yield 15.7%; 1H NMR (500 MHz, CDCl_3_) δ 7.48 (d, *J* = 7.9 Hz, 2H), 7.42 (d, *J* = 8.7 Hz, 2H), 7.33 (t, *J* = 7.8 Hz, 2H), 7.22 - 7.19 (m, 1H), 7.06 (d, *J* = 15.0 Hz, 1H), 6.92 (d, *J* = 15.0 Hz, 1H), 6.73 (d, *J* = 8.7 Hz, 2H), 2.99 (s, 6H). ^13^C NMR (125 MHz, CDCl_3_) δ 150.2, 138.3, 128.9, 128.7, 127.7, 126.8, 126.2, 126.0, 124.6, 112.6, 40.6. ^1^H NMR and ^13^C NMR spectra of (*E*)-N,N-dimethyl-4-styrylaniline were shown in Fig. S8.

**Fig. S8** ^1^H NMR and ^13^C NMR spectra of (*E*)-N,N-dimethyl-4-styrylaniline (6).

(*E*)-3-([1,1'-biphenyl]-2-yl)allyl acetate (7)


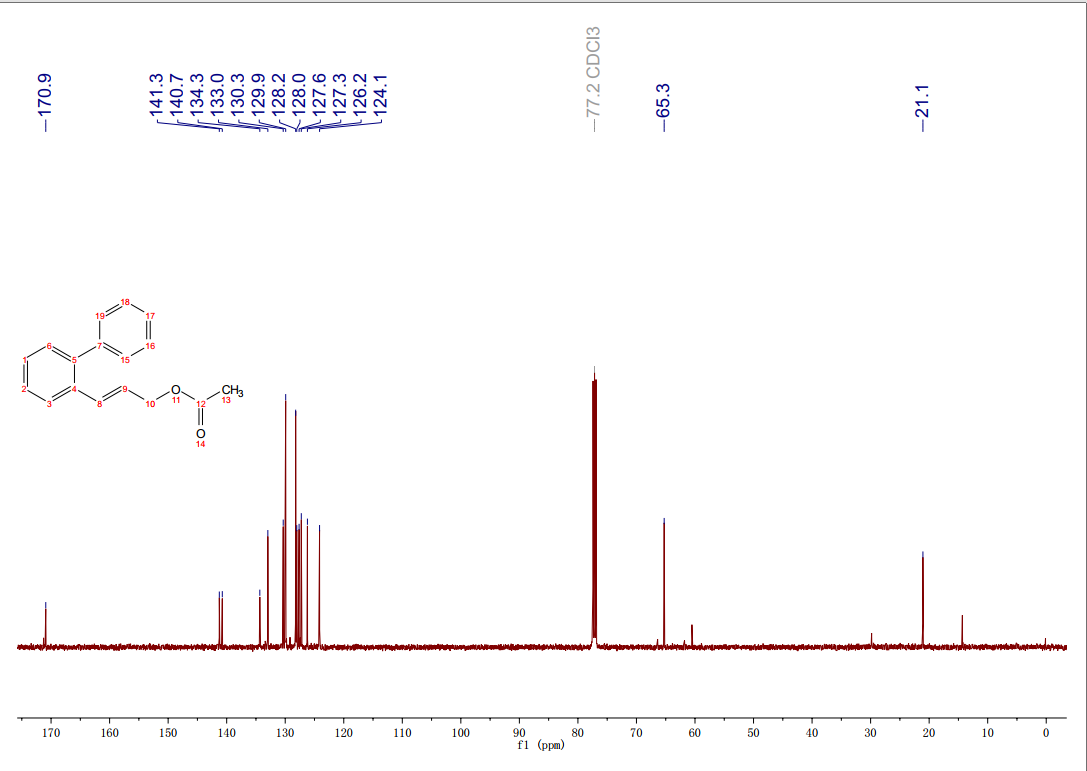

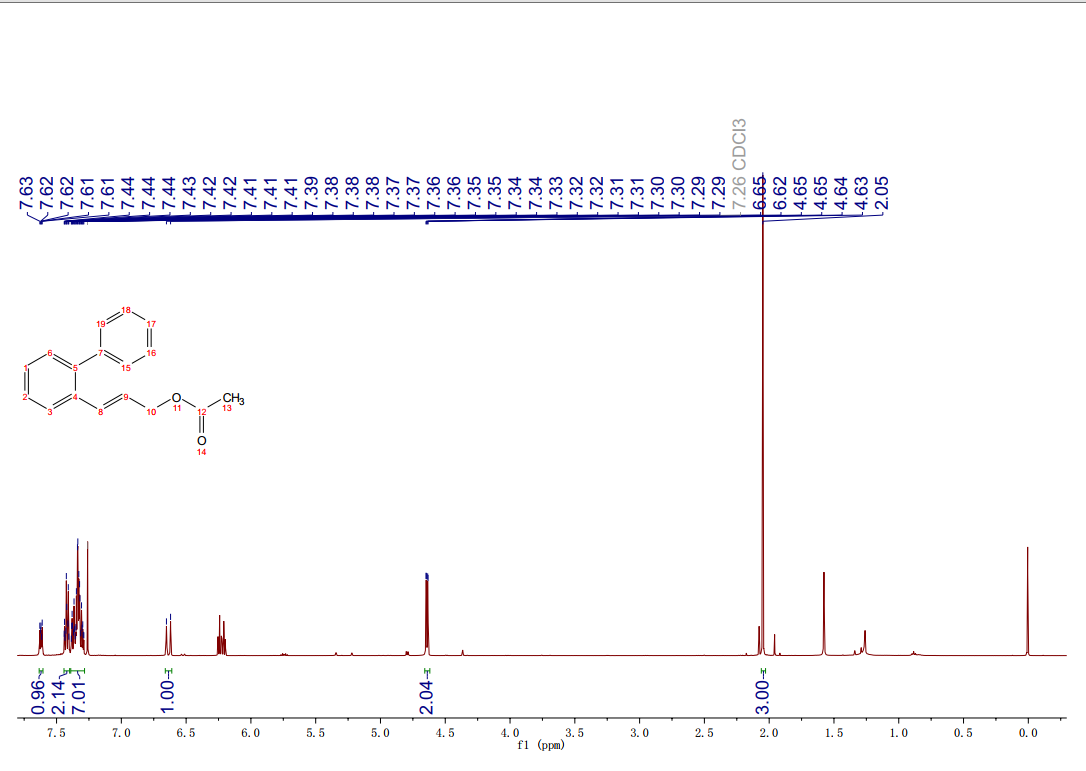
Yellowish oil, yield 13.1%; ^1^H NMR (500 MHz, CDCl_3_) δ 7.64 – 7.61 (m, 1H), 7.44 – 7.39 (m, 2H), 7.38 - 7.29 (m, 7H), 6.64 (d, *J* = 15.0 Hz, 1H), 4.64 (dd, *J* = 6.4, 1.4 Hz, 2H), 2.05 (s, 3H). 13C NMR (125 MHz, CDCl_3_) δ 170.9, 141.3, 140.7, 134.3, 133.0, 130.3, 129.9, 128.2, 128.0, 127.6, 127.3, 126.2, 124.1, 65.3, 21.1. ^1^H NMR and ^13^C NMR spectra of (*E*)-3-([1,1'-biphenyl]-2-yl)allyl acetate were shown in Fig. S9.

**Fig. S9** ^1^H NMR and ^13^C NMR spectra of (*E*)-3-([1,1'-biphenyl]-2-yl)allyl acetate (7).

Cyclohexyl (*E*)-3-(4-(tert-butyl)phenyl)acrylate (8)


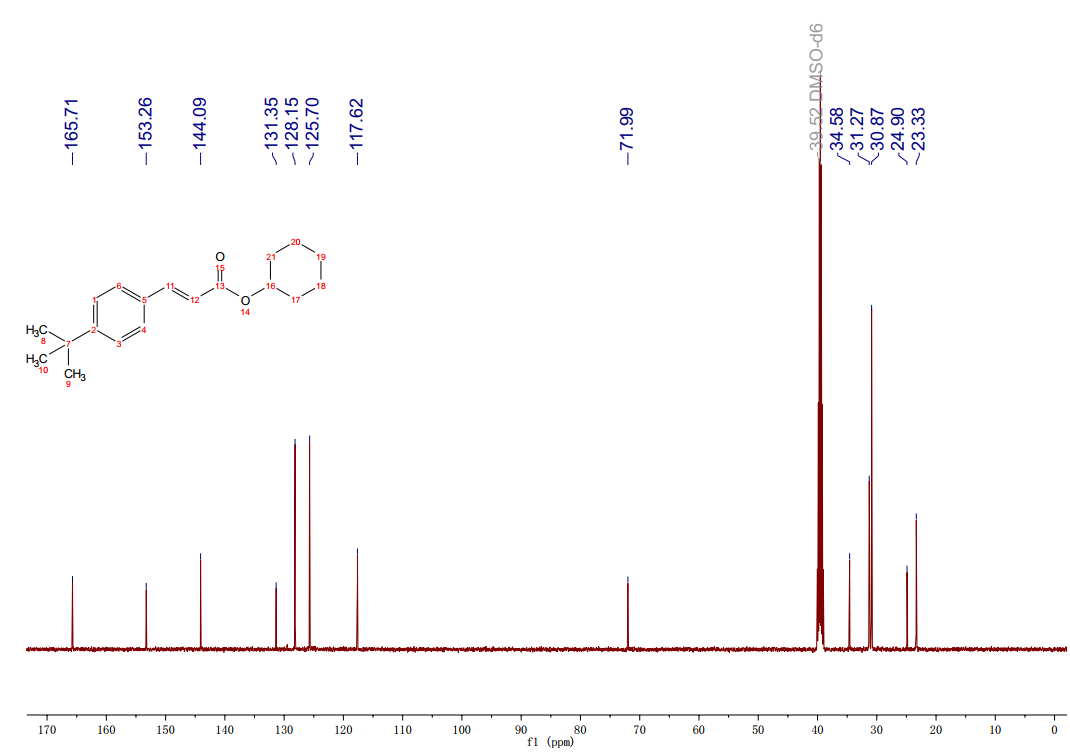

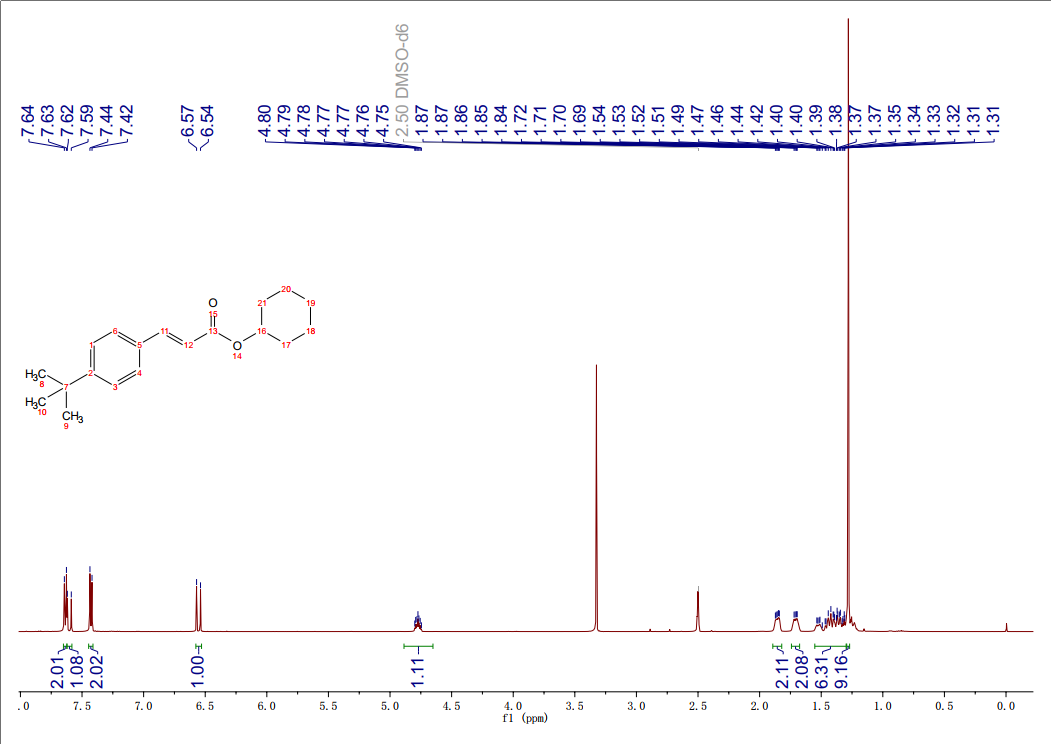
Colorless oil, yield 57.3%; ^1^H NMR (500 MHz, DMSO-*d*_6_) δ 7.64 (d, *J* = 8.4 Hz, 2H), 7.60 (d, *J* = 15.0 Hz, 1H), 7.43 (d, *J* = 8.4 Hz, 2H), 6.55 (d, *J* = 15.0 Hz, 1H), 4.80 - 4.75 (m, 1H), 1.87 - 1.84 (m, 2H), 1.72 - 1.69 (m, 2H), 1.54 - 1.31 (m, 6H), 1.28 (s, 9H); ^13^C NMR (125 MHz, DMSO-*d*_6_) δ 165.7, 153.3, 144.1, 131.3, 128.2, 125.7, 117.6, 72.0, 34.6, 31.3, 30.9, 24.9, 23.3. ^1^H NMR and ^13^C NMR spectra of cyclohexyl (*E*)-3-(4-(tert-butyl)phenyl)acrylate were shown in Fig. S10.

**Fig. S10** ^1^H NMR and ^13^C NMR spectra of cyclohexyl (*E*)-3-(4-(tert-butyl)phenyl)acrylate (8).

# Section S4 Table S1 to S3

**Table S1** Distribution of the reactions that don’t have chemical feasibility in the generated set.

| **Classification of reaction type** | **Amount** | **Rate (%)** |
| --- | --- | --- |
| intermolecular reaction | 2380 | 97.14 |
| intramolecular reaction | 70 | 2.86 |
| Total | 2450 | 100 |

**Table S2** Distribution of alkene reactants of the reactions that don’t have chemical feasibility in the generated set

| **carbon-carbon double bond classification of reactant** | **Amount** | **Rate (%)** |
| --- | --- | --- |
| Ethylene | 13 | 0.53 |
| Monosubstituted | 2370 | 96.74 |
| Disubstituted | 66 | 2.69 |
| Trisubstituted | 1 | 0.04 |
| Total | 2450 | 100 |

**Table S3** Distribution of halogenated aromatics and trifluoromethanesulfonate derivatives of the reactions that don’t have chemical feasibility in the generated set

| **Halogen atoms classification of reactants** | **Amount** | **Rate (%)** |
| --- | --- | --- |
| Cl | 113 | 4.61 |
| Br | 1683 | 68.69 |
| I | 599 | 24.45 |
| OTf | 55 | 2.24 |
| Total | 2450 | 100 |

# Section S4 Fig. S11 to S13

**
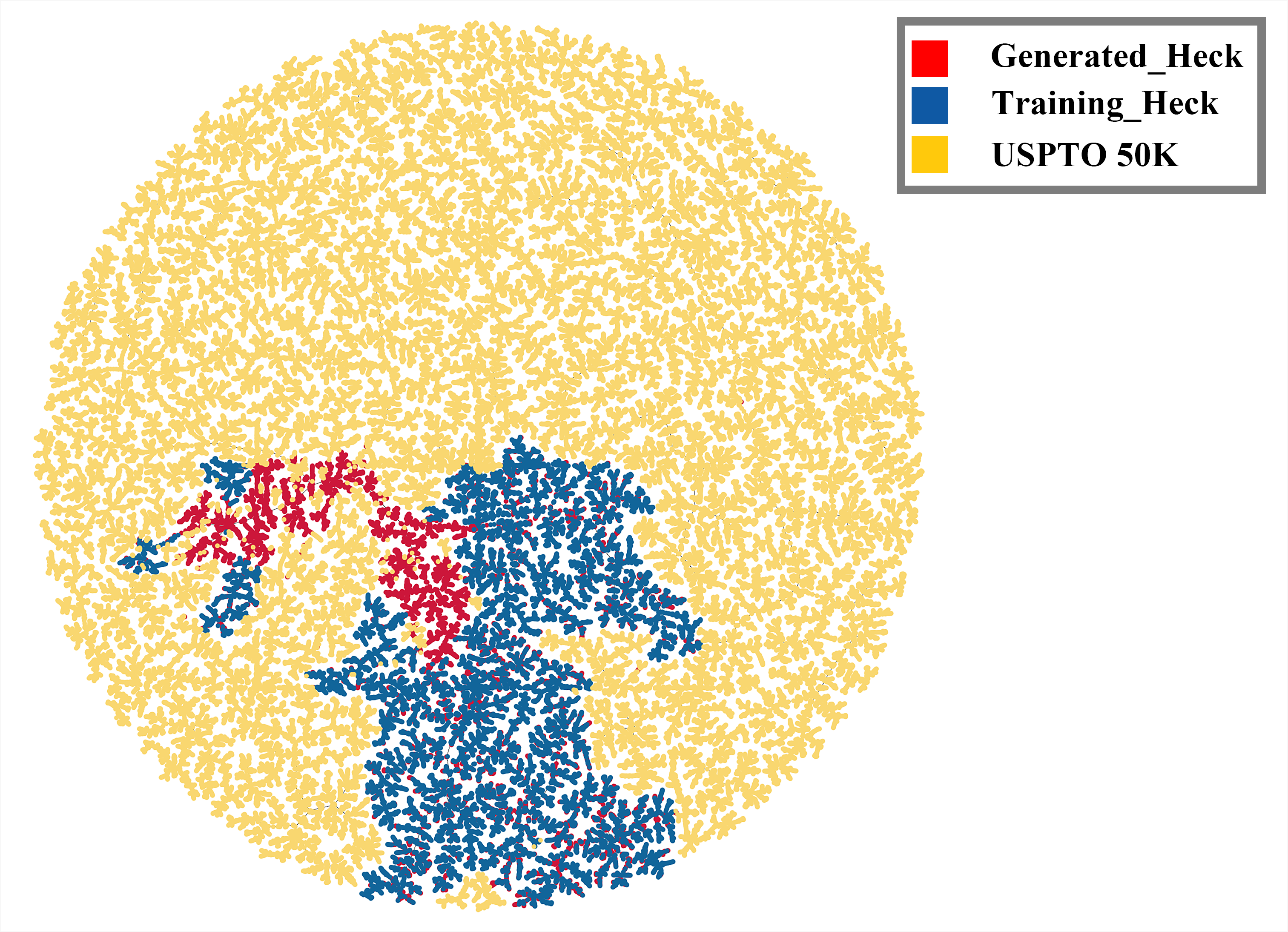
Fig. S11** The TMAP plot of reactions from training set (blue), generated novel reaction set (red) and USPTO 50K (yellow).

**Fig.S12** General mechanism of Suzuki reaction.

**Fig.S13** General mechanism of Kumada reaction.

# Section S5 Exploration of hyperparameters

Before generating the reactions with the Transformer-XL model, we first debugged the model. Therefore, we build a series of explorations based on training Heck reaction dataset to effectively select hyperparameters.

**Table S4**. The validity of the Transformer-XL model with different batch sizes. All are trained on a 1080 GPU and hidden_size = 512, drop_out = 0.1, n_head = 8, layer = 12.

| **Batch size** | **Validity^a^ (%)** | | |
| --- | --- | --- | --- |
|  | 1 | 2 | 3 |
| 64 | 90.2 | 90.2 | 90.0 |
| 128 | 90.2 | 90.0 | 90.1 |
| 256 | 90.0 | 89.7 | 89.6 |
| 512 | 90.1 | 89.7 | 90.0 |
| 1024 | 89.8 | 89.6 | 89.8 |

^a^The validity is the ratio of the reactions that all components are valid.

**Table S5**. The validity of the Transformer-XL model with different hidden sizes. All are trained on a 1080 GPU and batch_size = 64, drop_out = 0.1, n_head = 8, layer = 12.

| **Hidden size** | **Validity (%)** | | |
| --- | --- | --- | --- |
|  | 1 | 2 | 3 |
| 128 | 88.6 | 87.9 | 88.2 |
| 256 | 89.7 | 89.5 | 90.1 |
| 512 | 90.2 | 90.5 | 90.1 |
| 1024 | 90.1 | 89.7 | 90.2 |

**Table S6**. The validity of the Transformer-XL model with different drop out. All are trained on a 1080 GPU and batch_size = 64, hidden_size = 512, n_head = 8, layer = 12.

| **Drop out** | **Validity (%)** | | |
| --- | --- | --- | --- |
|  | 1 | 2 | 3 |
| 0.1 | 90.2 | 90.0 | 90.1 |
| 0.2 | 89.7 | 90.1 | 89.5 |
| 0.3 | 89.4 | 89.7 | 89.6 |

**Table S7**. Performance metrics for the different generative models: validity, uniqueness, novelty and availability.

| **Model** | **Validity** | | | **Uniqueness** | **Novelty** | **Chemical feasibility** |
| --- | --- | --- | --- | --- | --- | --- |
|  | Reactants | Products | All components |  |  |  |
| Transformer-XL | **91.64%** | **96.28%** | **90.20%** | 15.03% | 44.19% | **47.76%** |
| RNN | 84.03% | 78.18% | 69.58% | **97.94%** | **97.75%** | 4.73% |
| RNNAttn | 91.16% | 87.73% | 81.63% | 93.46% | 94.05% | 4.70% |

# Section S6 References

1. Amatore C, Carre E, Jutand, A and M'Barki MA (1995) Rates and mechanism of the formation of zerovalent palladium complexes from mixtures of Pd (OAc) 2 and tertiary phosphines and their reactivity in oxidative additions. Organometallics, 14: 1818-1826.

2. Jutand A and Mosleh A (1995) Rate and Mechanism of Oxidative Addition of Aryl Triflates to Zerovalent Palladium Complexes. Evidence for the Formation of Cationic (. sigma.-Aryl) palladium Complexes, Organometallics. 14: 1810-1817.

3. Thorn DL and Hoffmann R (1978) The olefin insertion reaction. J Am Chem Soc 100: 2079-2090.

4. Crisp GT (1998) Variations on a theme-recent developments on the mechanism of the Heck reaction and their implications for synthesis. Chem Soc Rev 27: 427-436.
